# Ribosomal protein uS7/Rps5 serine-223 in protein kinase-mediated phosphorylation and ribosomal small subunit maturation

**DOI:** 10.1038/s41598-018-19652-z

**Published:** 2018-01-19

**Authors:** Makoto Tomioka, Mitsugu Shimobayashi, Makoto Kitabatake, Mutsuhito Ohno, Yasunori Kozutsumi, Shogo Oka, Hiromu Takematsu

**Affiliations:** 1 Laboratory of Biological Chemistry, Human Health Sciences, Graduate School of Medicine, Kyoto University, Kyoto, Japan; 2 Laboratory of Membrane Biochemistry and Biophysics, Graduate School of Biostudies, Kyoto University, Kyoto, Japan; 3 Laboratory of RNA System, Institute for Frontier Life and Medical Sciences, Kyoto University, Kyoto, Japan; 4 Present Address: Biozentrum - Center for Molecular Life Sciences, University of Basel, Basel, Switzerland

## Abstract

Cellular translation should be precisely controlled in response to extracellular cues. However, knowledge is limited concerning signal transduction-regulated translation. In the present study, phosphorylation was identified in the 40S small subunit ribosomal protein uS7 (Yjr123w/previously called as Rps5) by Ypk1 and Pkc1, AGC family protein kinases in yeast *Saccharomyces cerevisiae*. Serine residue 223 (Ser223) of uS7 in the conserved C-terminal region was crucial for this phosphorylation event. S223A mutant uS7 caused severe reduction of small ribosomal subunit production, likely due to compromised interaction with Rio2, resulting in both reduced translation and reduced cellular proliferation. Contrary to optimal culture conditions, heat stressed *S223A* mutant cells exhibited increased heat resistance and induced heat shock proteins. Taken together, an intracellular signal transduction pathway involving Ypk1/Pkc1 seemed to play an important role in ribosome biogenesis and subsequent cellular translation, utilizing uS7 as a substrate.

## Introduction

Protein kinases can control cellular fates dependent on environmental cues; to enable fitness in the course of evolution. Since protein translation is regarded as the most costly cellular anabolic process^1^, its regulation is crucial for cells not to waste nutrients, concomitant with stress-resistance^2^. Ypk1 is a serine/threonine protein kinase in the AGC (protein kinases A, G and C) family in the budding yeast, *Saccharomyces cerevisiae*^3^. Ypk1 plays a crucial role in signal transduction affecting cellular proliferation^4^, sphingolipid-mediated signal transduction^5^, endocytosis^6^ and protein translation^7^. However, limited information is available regarding Ypk1’s target substrates to explain these multiple roles.

Among the yeast AGC protein kinase family, Ypk1 exhibits the strongest sequence similarity to Ypk2, both of which are thought to be paralogous, and related to mammalian SGK^4^. Pkc1^8^ and Sch9^9^ form a subfamily, which may be related to the mammalian PKC family. In relation to ribosomal protein phosphorylation, Ypk3, another AGC kinase family member, was shown to be orthologous to mammalian S6 kinase in yeast^10,11^ downstream of TOR complex 1 (TORC1) signaling to phosphorylate ribosomal protein S6. Ypk1 was proposed to function in translation, independent of TORC1^7^, and its loss causes a slow growth phenotype^3^. Currently, information is limited regarding protein kinase-substrates, which confound full understanding of the relationships of signal transduction pathways.

To search for downstream event(s), proteomic screening utilizing *ypk1∆* cells was conducted in the present study. From this analysis, ribosomal 40S small subunit (SSU) protein uS7/Rps5 was identified as a potential downstream target molecule. uS7 is an essential SSU ribosome protein (R-protein), which is required for early ribosome biogenesis. In the ribosome biogenesis, ribosomal DNA is transcribed by RNA polymerase I and rRNA is subsequently processed during maturation of ribosomes to form 18S, 5.8S and 25S rRNA^2^. A late step in 40S SSU maturation occurs in the cytosol where 43S pre-small subunit interacts with a protein complex comprising kinase-like Rio2 and nuclease Nob1 and others^12^. Deletion of *uS7* results in early ribosomal biogenesis defect, that cause depletion of ribosomes in cytoplasmic space^13^. In addition, it is reported that both N- and C-terminus seemed to be important for uS7 because truncation of either region causes translational defects^14–16^. C-terminal part of uS7 could serve as a docking site for Rio2^16^. Phosphorylation from casein kinase II activity seemed to play important role in ribosomal trafficking in mammalian systems^17^. More recently, it was reported that point mutation of amino acid residues in the C-terminal portion of uS7 could be involved in regulation of start codon scanning of *GCN4*^18^ because this region could regulate the interaction with eIF2α as a ternary complex^19^. These reports indicated that post-translational modification of uS7 could be an important cellular event.

In the present investigation, the relationship between Ypk1 and uS7 was studied in the context of protein phosphorylation. Ypk1 and uS7 showed intracellular interaction and Ypk1 utilized uS7 as a substrate. Point mutation of uS7 showed that Serine 223 (Ser223) could be the important determinant for a kinase substrate since S223A (alanine mutation) caused attenuated phosphorylation by Ypk1 and Pkc1. Cells expressing uS7-S223A showed reduced SSU implicating Ser223 as important for ribosome biogenesis. Unlike overall translation under optimal conditions for yeast, heat stress-dependent Hsp12, Hsp30 and Ssa4 production was augmented in *S223A* cells, indicating that the *S223A* mutation caused skewed preference in translation. These were novel observations suggesting possible protein kinase regulation in ribosome biogenesis and the still-elusive target-dependent translational event(s)^20^.

## Results

### uS7 identified as a Ypk1 downstream molecule

**Figure 1:**
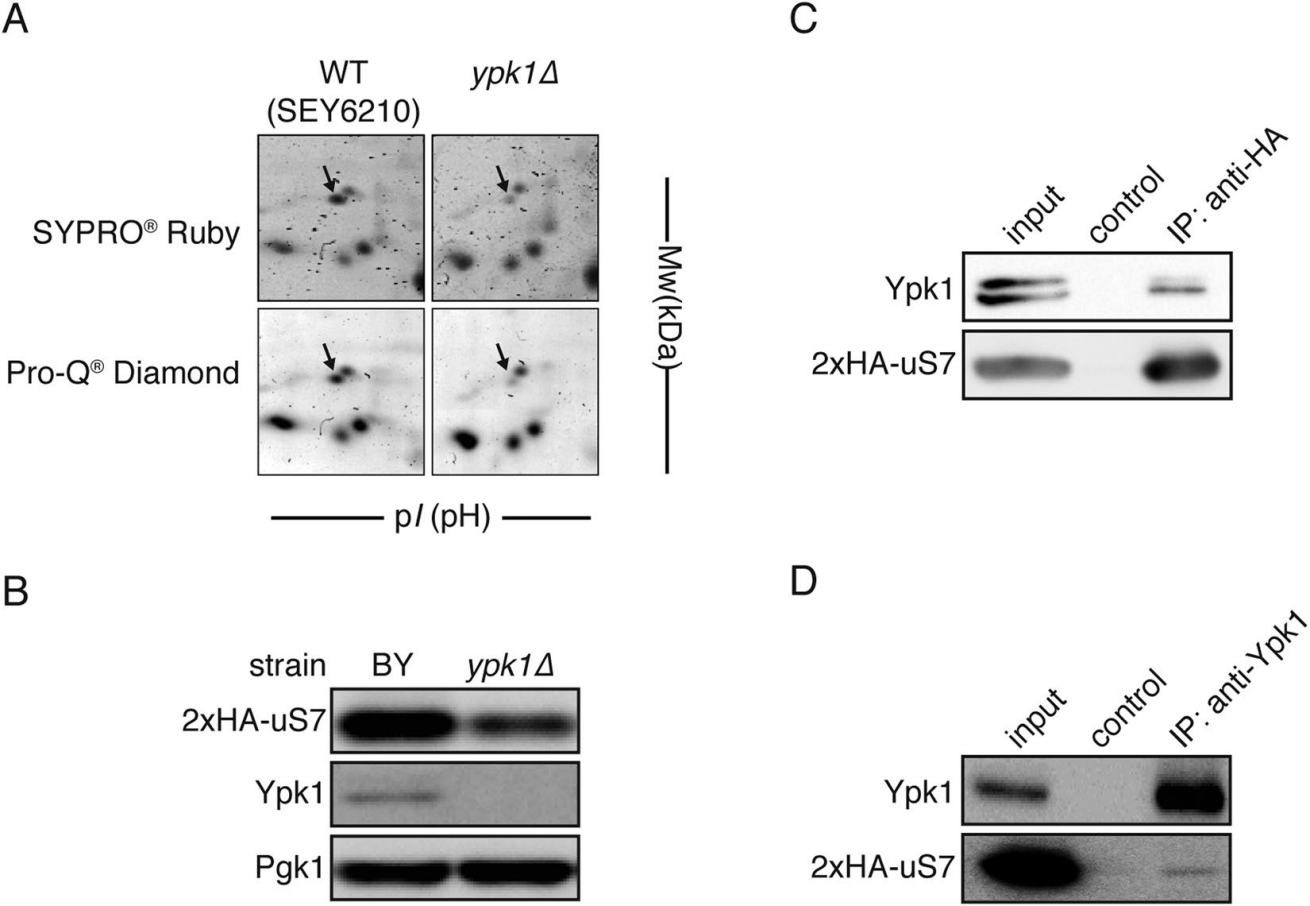
Ribosomal protein uS7 abundance is affected in *ypk1∆* strain. (**A**) Cytosolic proteins extracted from WT and *ypk1∆* strain were separated by 2D-gel. Protein spots were visualized with SYPRO Ruby (for protein abundance) and Pro-Q Diamond (for protein phosphorylation). From each strain, pI values around 5 and molecular weight around 30 kDa is shown. Whole image is available in Supplemental Fig. S1. Arrows indicate the spot of interest, the staining signal of which was suppressed in *ypk1∆(SEY6210)*. (**B**) Reduced expression levels of uS7 in *ypk1∆* strain. Plasmid-derived uS7 level was monitored in *ypk1∆* cells. Cells harboring single copy expression plasmids in logarithmic phase were harvested and lysate was prepared. Protein extract (5 μg/lane) was analyzed with Western blotting. uS7 was detected by anti-HA. Ypk1 was detected by anti-Ypk1. Anti-Pgk1 was utilized as loading control. (**C**,**D**) Physical interaction of Ypk1 and uS7. 2xHA- uS7 (**C**) or Ypk1 (**D**) was immunoprecipitated with anti-HA or anti-Ypk1 antibody with protein G-Sepharose and co-precipitation was examined with Western blotting.

Protein kinase Ypk1 could be involved in many crucial cellular events such as cellular proliferation, endocytosis and cellular sphingolipid signaling^3,5,6,21^. By screening proteins affected in a *ypk1∆* strain, potential multiple downstream events in Ypk1 signaling function could be studied. Proteins were extracted from *ypk1∆* cells and their abundance compared with control cells on 2D-PAGE gel analysis using protein fluorescence staining with SYPRO Ruby dye (Supplemental Table S1, Supplemental Fig. S1). A protein spot with pI = 5, MW = 30 kDa on the 2D-PAGE gel was pinpointed, where intensity was attenuated in *ypk1∆* cells (Fig. 1A). When phospho-protein staining dye, Pro-Q Diamond was used, consequent reduction of this spot was seen in *ypk1∆ cells* (Fig. 1A). Proteomic MS-analysis indicated that this spot was identified as uS7. uS7 is an essential protein of SSU located adjacent to the ribosome E site (Supplemental Fig. S2). When HA-tagged uS7 was expressed and detected with Western blotting, the uS7 level was attenuated by roughly 40% in the *ypk1∆* strain (Fig. 1B). Therefore, uS7 level appears to be modulated by Ypk1. To further examine the relationship of Ypk1 with uS7, their protein-protein interaction was assessed. When HA-uS7 was immunoprecipitated, endogenous Ypk1 was co-immunoprecipitated (Fig. 1C). Likewise, Ypk1 immunoprecipitate contained HA-uS7, although in this case, HA-uS7 was faintly detected (Fig. 1D). This result indicated that only a limited portion of uS7 is physically associated with Ypk1. Thus it is not likely that uS7 and Ypk1 form a fixed complex, rather the interaction could be transient.

### Serine 223 is important for uS7 function

**Figure 2:**
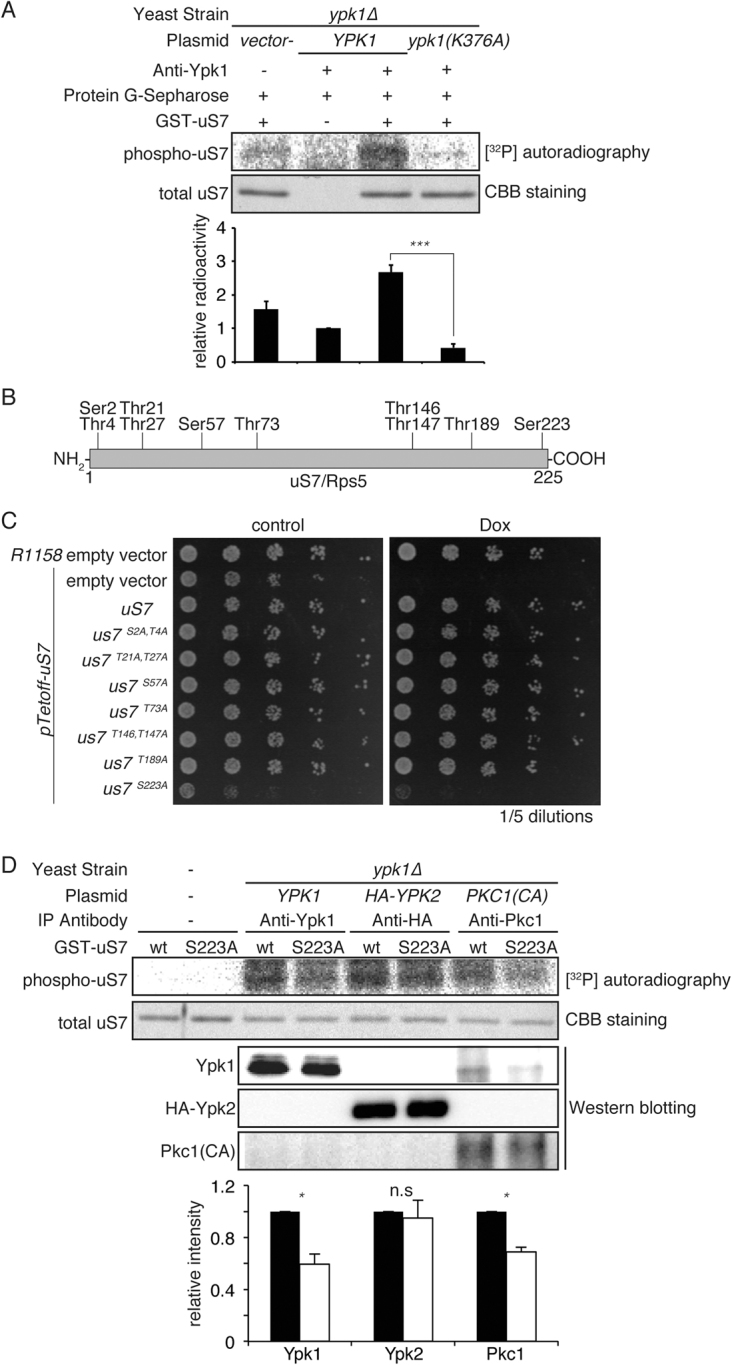
Phosphorylation of uS7 by Ypk1. (**A**) uS7 phosphorylation *in vitro* by Ypk1. Logarithmically growing yeast cells were harvested and Ypk1 was immunoprecipitated by anti-Ypk1 and Sepharose-conjugated protein G. GST-conjugated uS7 was expressed in *E. coli* and purified by glutathione-Sepharose. Ypk1 containing resin was suspended with uS7 and incubated with [γ-^32^P]-ATP as described in experimental procedures. Post-incubation samples were separated on SDS-PAGE gel and radioactivity was measured by exposing to an image plate for BAS-2500. Mean relative kinase activity was plotted from triplicate experiments with SD error. Statistical significance was assessed by Student’s *t*-test. The triple asterisk denotes that *p* is less than 0.001. To examine the background level of assay, lysate prepared from *ypk1∆* strain with empty vector was used. (**B**) Schematic presentation of uS7 phosphorylation site candidates. Open reading frame of uS7 is depicted as a rectangle. Potential serine or threonine residues identified by NetPhosYeast 2.0 software are expressed as vertical lines. (**C**) Alanine mutation of uS7. A series of alanine mutations were introduced to phosphorylation candidate sites. Mutant uS7s were expressed from plasmid vector pRS415 with its own promoter whereas endogenous uS7 was turned off utilizing Tet-OFF with doxycycline. Serial dilutions of indicated yeast cells were spotted onto SD plate and incubated at 30 °C with or without 10 μg/mL doxycycline (Dox). (**D**) Involvement of Pkc1 but Ypk2 in uS7 phosphorylation. *In vitro* phosphorylation assay was carried out as Fig. 2A except Ypk2 and Pkc1 was also utilized as a kinase source and S223A (uS7-S223A) mutant was also utilized as substrate. Mean relative kinase activity was plotted from triplicate experiments with SD error. Statistical significance was assessed by Student’s *t*-test. The single asterisk denotes that *p* is less than 0.05.

The relationship between Ypk1 and uS7 could be a protein kinase and its substrate, thus this hypothesis was assessed, although loss of ProQ diamond staining may not account for the loss of specific phosphorylation, due to the obvious reduction of uS7 level (Fig. 1A). When recombinant GST-tagged uS7 was prepared in *E. coli* and Ypk1 kinase prepared in yeast, Ypk1(WT) phosphorylated uS7 in the kinase assay whereas a kinase inactive mutant of Ypk1-K376A(KD) did not (Fig. 2A), indicating uS7 to be an Ypk1 substrate. Due to the presence of background phosphorylation of uS7 in control conditions (lysate with protein G-Sepharose), we also utilized GST-purified Ypk1, which has been used in large scale analyses of Ypk1 target^22^. GST-purified Ypk1 also consistently enhanced uS7 phosphorylation by Ypk1 (Supplemental Fig. S3). It has been suggested that Ypk1 has a substrate specificity toward peptide motif RxRxxS/T in which R, x, S and T represent arginine, any amino acid, serine and threonine, respectively^4,23^. However, there was no motif matching this sequence within uS7, although some phosphorylation sites are previously listed in the proteomic studies^24,25^. To determine potential phosphorylation sites by protein kinases, this consensus sequence for phosphorylation was screened with the help of software NetPhosYeast and NetPhos 2.0 (Technical University of Denmark, http://www.cbs.dtu.dk/services/NetPhos/), which identify potential phosphorylation sites (Fig. 2B). A series of alanine mutants was made to screen potential phosphorylation events at candidate sites, and to determine crucial site(s) for uS7 function. Since *uS7* is an essential gene, a TET-OFF promoter-knock-in strain was utilized to shut off endogenous uS7 only when required, and rescued with mutant uS7s from a single-copy plasmid bearing its own promoter. Mutant uS7 function can be measured in a proliferation assay upon doxycycline addition to suppress endogenous uS7, as this compound turns off TET promoter transcription to reduce this essential protein only when needed. Among the series of mutants, only an S223A mutation within the C-terminal portion at serine residue 223 (Ser223) exhibited attenuated growth (Fig. 2C), indicating phosphorylation at this site may be important for uS7 function. Consistent with this result, it was recently reported that an *S223D* mutation could result in an increase of 40S/60s ratio^19^. Computer-based NetPhos 2.0 screening suggested that Ser223 is a potential phosphorylation site for protein kinase C (PKC), also a member of the AGC protein kinase family, similar to Akt and SGK. A stronger growth defect was found when cells were rescued with both *S223D* and *S223E* mutant, indicating that phosphorylation at this site needs to be properly regulated, or S223D/E mutation cannot mimic phosphorylated serine (Supplemental Fig. S4). It was recently reported that Ser223 mutations could cause conformational change of eIF2a-uS7 interaction, where S223A forms “open” and S223D “closed” states^19^. Therefore, phosphorylation at this site could regulate cellular translational initiation. Alternatively, any point mutation at this site is not tolerated for optimal growth.

### Effect of S223A mutation as kinase substrate

When S223A mutant recombinant uS7 was used as a substrate for an *in vitro* kinase assay, phosphorylation from Ypk1 was severely attenuated to the control level, indicating that this site is directly phosphorylated by Ypk1 (Fig. 2D). Ypk2 is regarded as paralogous with Ypk1. Ypk2 also phosphorylated uS7, indicating that both kinases can redundantly phosphorylate uS7 (Fig. 2D). However, S223A did not exhibit statistically significant attenuation of the phosphorylation by Ypk2 in repeated analyses (Fig. 2D), indicating that Ypk2 may phosphorylate uS7 at a different site(s) than Ser223. Pkc1 is another AGC kinase in yeast^26^, which exhibits sequence similarity with Ypk1 and Ypk2 especially in the protein kinase domain, and is essential for cell survival. Since PKC involvement was suggested at this site in computer-based screening, Pkc1 was also used as enzyme source. uS7 was phosphorylated and the phosphorylation signal was attenuated in the S223A mutant (Fig. 2D) as predicted by *in-silico* screening. Therefore, Ser223 could be phosphorylated by multiple AGC family protein kinases including Ypk1 and Pkc1 but not Ypk2. This is the first substrate protein which Ypk1 and Ypk2 could differentially phosphorylate.

### Attenuated overall translation upon *S223A* mutation

**Figure 3:**
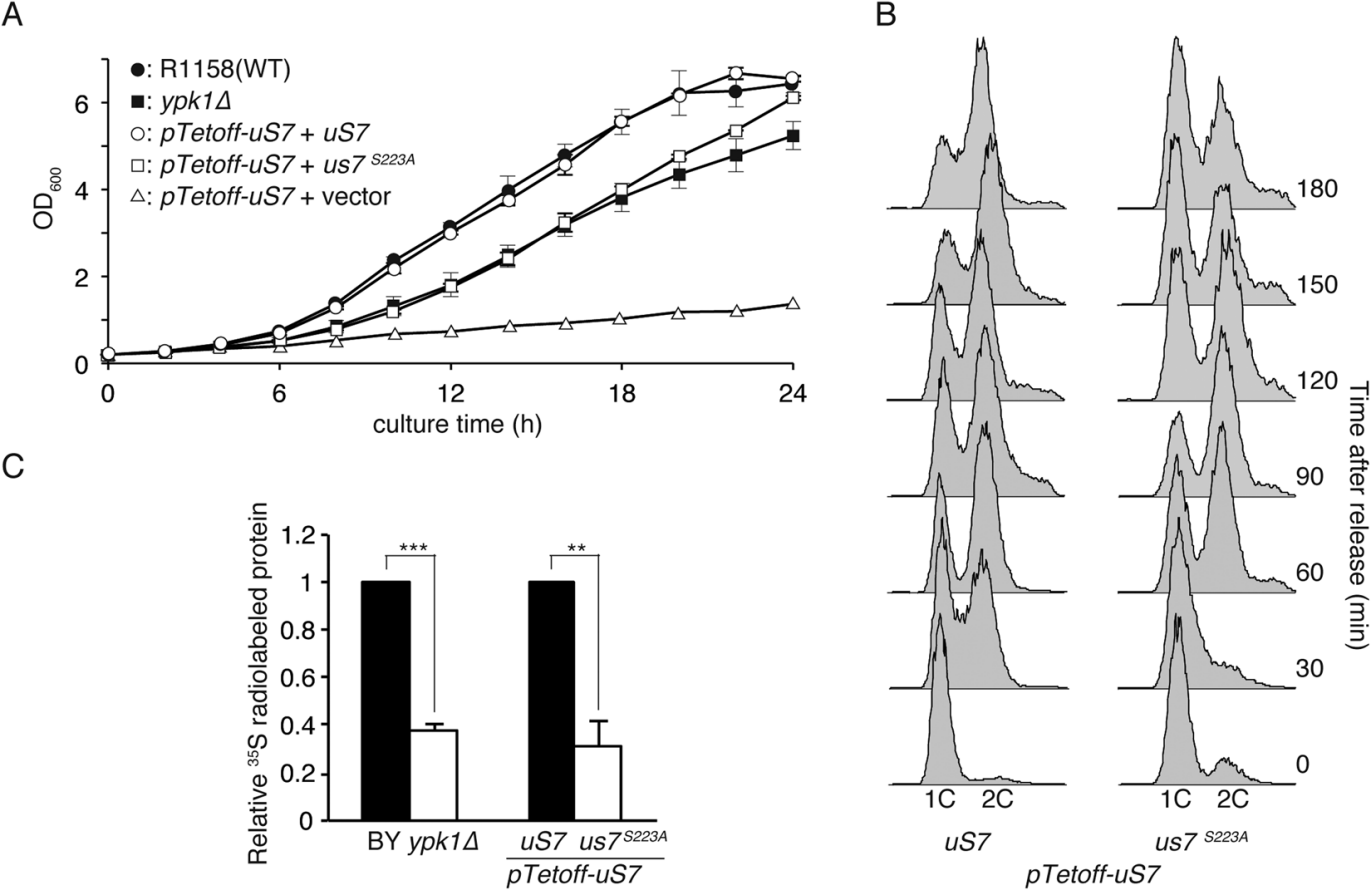
S223A mutation of uS7. (**A**) Growth curve of uS7 mutant strains. uS7 or its mutant was expressed from plasmid vector in Tet-OFF knockin strain for induced uS7 depletion from cells. Time course of cellular proliferation was measured in liquid culture by monitoring OD_600_ values. (**B**) Cell cycle progression of *S223A* mutant strains. Yeast cells with indicated genetic background was exposed to α-factor to induce G_1_-arrest of the cell cycle. Cells were washed with medium to release the cell cycle. DNA content in each cell was measured by the combination of PI staining and flow cytometric detection after RNase digestion. Cell cycle progression was monitored by the increase of 2-copy (2 C) cells. (**C**) Protein translation in *S223A* mutant. Yeast cells of indicated genotype were metabolically labeled with [^35^S] Met-Cys and cellular protein was recovered with TCA-precipitation. Radioactivity incorporated in *de novo* protein synthesis and protein abundance in the TCA-precipitate was measured by liquid scintillation counter and Bradford method, respectively. *De novo* protein translation in each strain was deduced by examination of radioactivity that was normalized with proteins concentration. Mean relative values were plotted from three independent experiments. Statistical significance between groups was assessed by Student’s *t*-test. The double asterisk denotes that *p* is less than 0.005, and the triple asterisk denotes that *p* is less than 0.001.

Effect of *S223A* mutation of uS7 on cellular proliferation was monitored. Both the *S223A* mutant and *ypk1∆* cells exhibited slower proliferation in liquid culture (Fig. 3A). This slow proliferation of the *S223A* mutant was accounted for by a longer lag-phase. When the cell cycle was arrested by α-factor and released, WT cells start to enter S-phase as early as 30 min whereas *S223A* cells start chromosome duplication after 60 min (Fig. 3B). uS7 is an R-protein and Ypk1 is also important for protein translation^7^, thus, consequences of *S223A* mutation for translational activity was monitored by means of radioactive ^35^S amino acid(s) incorporation measurement into freshly translated proteins. In *S223A* mutant cells, protein translation was attenuated by roughly 60% (Fig. 3C), the rate of which was similar in the *ypk1∆* strain (Fig. 3C). This reduction is not caused by differences in radioactive amino acid uptake in the metabolic labeling experiments, because cells were equally radiolabeled within 20 min (Supplemental Fig. S5).

### Effect of *S223A* mutation on ribosomes

**Figure 4:**
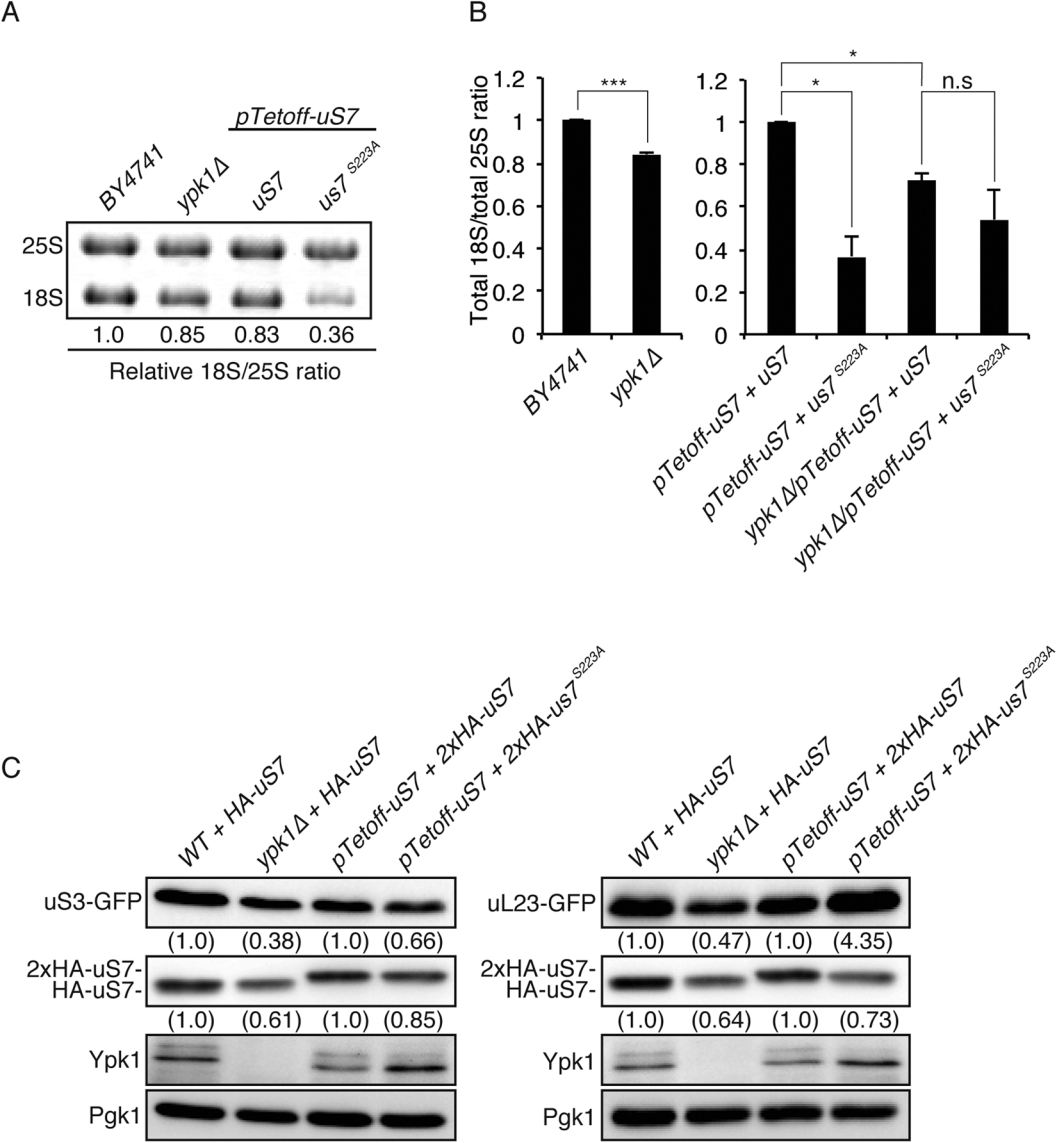
Reduced 18S ribosomal small subunit in *S223A* mutant uS7. (**A**,**B**) Attenuated 18S rRNA. Total RNA was purified from *ypk1∆* cells and *us7* mutant cells and analyzed by agarose gel electrophoresis and real-time PCR. Ratio of 18S and 25S was calculated from relative fluorescence intensity. (**A**) rRNA abundance was also examined by real-time PCR. (**B**) Statistical significance was assessed by Student’s *t*-test. The asterisk indicates that *p* is less than 0.05, and the triple asterisk denotes that *p* is less than 0.001. (**C**) Small subunit-specific reduction with *S223A* mutation and non-specific ribosomal protein reduction in *ypk1∆* cells. Ribosomal protein abundance was monitored in *ypk1∆* cells and *S223A* mutant cells harboring *uS3-GFP* or *uL23-GFP*. Cells in logarithmic phase were harvested and lysate prepared. Protein extract was analyzed with Western blotting. Both uS3 and uL23 were detected by anti-GFP and uS7 detected by anti-HA. Ypk1 was detected by anti-Ypk1. Anti-Pgk1 was utilized as loading control. Relative chemiluminescent values in each band are compared to isogenic controls.

Given the compromised translational activity of *ypk1∆* and *S223A* cells, reduction in uS7 protein level may account for the systemic ribosomal defect, rather than a specific event between Ypk1 and uS7. To evalutate this, rRNA abundance was examined in *S223A* and *ypk1∆* cells. When total RNA was run on agarose gel, 18S rRNA was reduced in the *ypk1∆* and a more prominent reduction was noted in *S223A* cells (Fig. 4A). This reduction was also confirmed by real-time PCR experiments (Fig. 4B, Supplemental Fig. S6). Attenuated 18S rRNA in the *S223A* mutant could be related to Ypk1-mediated phosphorylation because a subtle reduction of 18S rRNA was also noted in *ypk1∆* cells (Fig. 4A). *S223A*-mediated 18S reduction was not significant in the *ypk1∆* background (Fig. 4B), further supporting that Ypk1 and uS7 are involved in a similar genetic pathway. Protein level of uS7 was also suppressed with both in *ypk1∆* and *S223A* mutations when compared to respective controls (Fig. 4C). Whole 40S SSU seemed to be affected in *S223A* cells because uS3/Rps3, another SSU R-protein, was also reduced (Fig. 4C), suggesting the reduction was not specific to uS7. Nucleolar production of SSU can be compromised in *S223A* cells. However, S223A protein is localized in the cytoplasmic space in microscopic observation, indicating that S223A is incorporated into pre-small subunit, exported and remains in cytoplasmic space (Supplemental Fig. S7). These data suggested that subsequent maturation steps could be affected in *S223A* cells. Ribosomal large subunit (LSU) protein was also examined to understand ribosomal maturation in *S223A* cells. Crucially different from SSU-restricted reduction in *S223A* cells, *ypk1∆* cells also exhibited loss of uL23/Rpl25, an LSU component. Because the same protein amount was loaded, uL23 seemed to be overrepresented in *S223A* cells. Thus *ypk1∆* cells suppressed both large and small subunit proteins (Fig. 4C) whereas *S223A* cells suppressed only SSU proteins. Simultaneous loss of SSU and LSU in *ypk1∆* cells could explain very mild suppression of 18S rRNA because the same amount of RNA was loaded onto the gel (Fig. 4A–B), which causes compensation in *ypk1∆* cells. These data collectively indicate that *ypk1∆* cells have a combination SSU defect, similar to the *S223A* mutation, in addition to the LSU defect.

### Loss of mature 40S small subunit and polysomes in *S223A* cells

**Figure 5:**
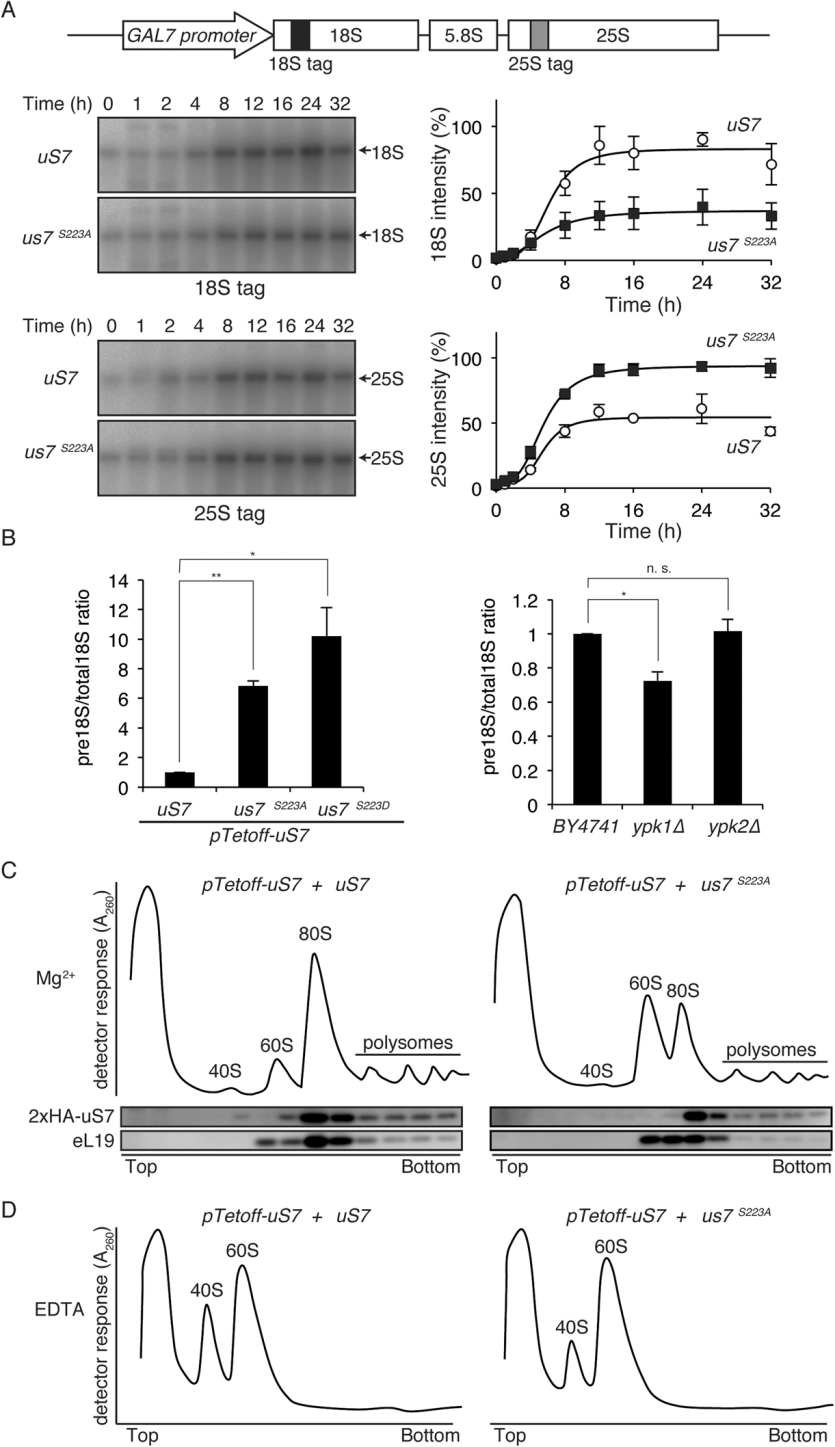
Maturation defect of SSU in *S223A* cells. (**A**) Reduction of 18s rRNA expression in *S223A*. Ribosomal DNA mini gene with *GAL7* promoter and tag sequence for probing is schematically represented (top). Ribosomal RNA transcription was activated in galactose-containing media. After indicated time with galactose, cells were harvested and total RNA purified. RNA samples were analyzed with Northern blotting utilizing tagged probes for 18S and 25S rRNA (arrows), which could detect only mini gene-derived rRNA species as described in experimental procedures. Time course change of 18S and 25S rRNA was plotted from triplicate experiments. (**B**) Change of the pre-18S rRNA. Total RNA was purified from *uS7*, *us7-S223A*, *us7-S223D*, *BY4741*, *ypk1∆* and *ypk2∆* cells and quantitatively amplified using indicated primer to monitor total rRNA and unprocessed pre-18S rRNA, respectively. Fold change of the ratio of pre-18S to total 18S is compared to control conditions. Statistical significance was assessed by Student’s *t*-test. The asterisk indicates that *p* is less than 0.05, and the double asterisk denotes that *p* is less than 0.005. (**C**,**D**) Polysome analyses of *S223A*. Yeast cells were harvested and lysate from indicated strains ultracentrifuged in sucrose density gradient sedimentation. After centrifugation, samples were recovered from the top of the ultracentrifuge tubes and change in A_260_ value was recorded by on-line detector to visualize RNA. Peaks for 40S, 60S, 80S ribosomes and polysomes were indicated (Mg^2+^) based on the presence of uS7 and eL19 proteins in fractions judged from Western blotting (**C**). To analyze 40S and 60S ribosome abundance, lysate was treated with EDTA to dissociate 80S and polysomes prior to ultracentrifugation (EDTA) (**D**).

Reduced level of SSU could be caused by aberrant ribosome biogenesis. To eliminate the possibility of *rDNA* transcription change by RNA polymerase I, a *GAL7* promoter-based *rDNA* construct was utilized to examine ribosome production in *S223A* cells (Supplemental Fig. S6)^27^. When galactose was added to the medium, both 18S and 25S rRNA were induced. The induction time course from *GAL7* promoter was measured for 18S and 25S rRNA in Northern blotting with a tagged sequence (Fig. 5A). Under this condition, *S223A* cells had attenuated 18S rRNA (Fig. 5A). In contrast, induction of 25S rRNA was detected (Fig. 5A), which may be compensatory because identical amounts of RNA (1.6 μg/lane) were loaded on the gel. It is notable that even though 18S was attenuated, unlike cells with C-terminal 7 amino acid deletion mutant uS7, uncleaved 35S and 20S rRNA were not obviously enriched in *S223A* cells (Fig. 5A)^16^. When the ratio of pre-18S rRNA species to mature 18S was measured using a primer set (Supplemental Fig. S6), an increased pre-18S ratio was detected (Fig. 5B) in *S223A* cells, indicating that mature 40S SSU is reduced in *S223A* cells. *S223A* and *S223D* mutant exhibited similar suppression of 18S rRNA and when pre-18S and total 18S were compared. Unexpectedly, this ratio was slightly increased in *ypk1∆* cells (Fig. 5B); the reduction mechanism of 40S SUU could be subtly different in *S223A* and *ypk1∆* cells (Fig. 4A,B), although this is not yet understood.

These results suggested that 18S rRNA processing is affected in *S223A* cells at least at the level of purified rRNA. Prompted by these results, direct examination of ribosomes was carried out. Using sucrose gradient sedimentation to separate ribosomes according to their densities, WT showed the following order of peaks of A_260_ absorbance; free-RNA, 40S, 60S, 80S and polysomes as indicated by the presence of uS7 and eL19/Rpl19a/b (Fig. 5C). In the *S223A* mutant, severe reduction of both 80S and polysome fractions was noted (Fig. 5C). Instead, 60S ribosomes were increased in these cells (Fig. 5C). Treatment of ribosomes with EDTA results in dissociation of subunits, where a clear reduction of 40S ribosomes was observed in the *S223A* cells (Fig. 5D). From these results, we concluded that S223A mutation results in attenuated 40S ribosome maturation. These results were consistent with a previous report regarding the C-terminus truncation of uS7, which showed that deletion of C-terminal 7 amino acids is required for proper SSU maturation. Although *ypk1∆* cells did not result in specific loss of 18S rRNA (Fig. 4A,B) and SSU R-proteins (Fig. 4C), 18S maturation was affected in comparison with pre-18S and total 18S (Fig. 5B). When *ypk1∆* cells were examined, a minor reduction of 80S ribosomes was detected with similar overall 40S/60S ratio as found in EDTA-treated sample (Supplemental Fig. S8). Given that translation was deficient (40% of WT control (Fig. 3C)) in the *ypk1∆* cells, these data indicated that *YPK1* deletion causes deficiency in both 40S and 60S subunits.

### Possible involvement of Rio2 in the phenotype

**Figure 6:**
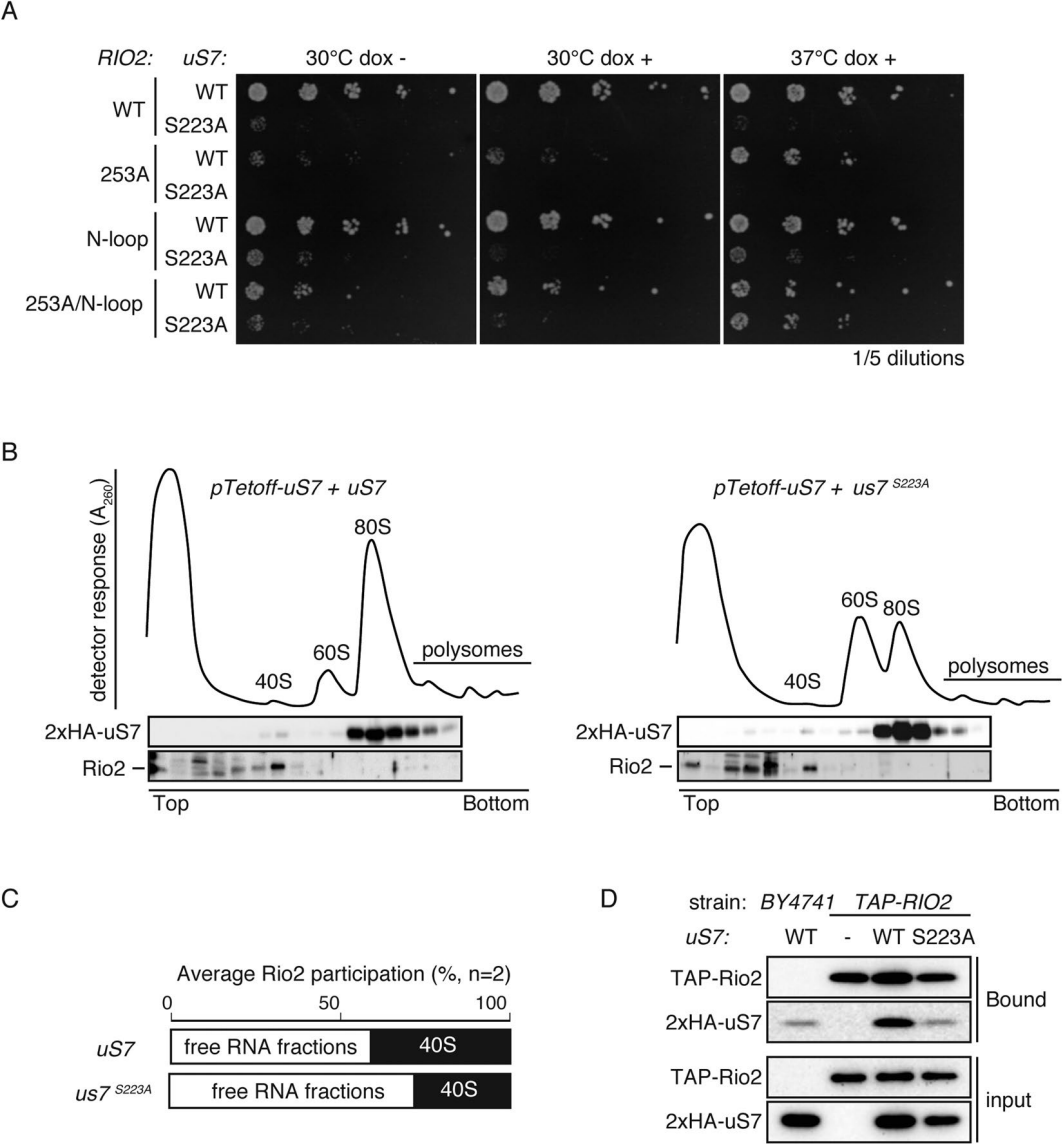
Relation of *S223A* mutation and SSU maturation gene. (**A**) Genetic mutant interaction of *uS7* and *RIO2*. Heat stress resistance phenotype was examined in the genetic background carrying *uS7* mutation and *RIO2* mutation. Tet-OFF promoter-harboring *uS7* cells were rescued with a single copy plasmid encoding *WT uS7* or *S223A* and *WT RIO2* or mutated *rio2*. (**B**) Loss of Rio2 protein in the ribosome upon mutation of *uS7*. Participation of Rio2 in the ribosomes of *S223A* mutant was examined with sucrose gradient ultracentrifuge sedimentation assay of ribosomes. After in-line monitoring of RNA (A_260_ values), samples were fractionated and analyzed by Western blotting using anti-HA (to observe uS7) and anti-Rio2. (**C**) Loss of 40S-interacting Rio2 in *S223A*. Rio2 participation in the 40S-containing fractions (filled box) was measured by densitometry of Western blotting in Fig. 6B and ratio to the Rio2 level in the free-RNA fraction (open box) is expressed as a percentage in duplicate experiments. (**D**) Decreased interaction between Rio2 and uS7 upon *S223A* mutation. Physical interaction of Rio2-uS7 was monitored by co-precipitation of TAP-tagged Rio2 with HA-tagged WT or S223A uS7 (Bound). Lysate (input) expressed TAP-Rio2 was precipitated with IgG-Sepharose. uS7 co-precipitation to Rio2 was examined with Western blotting. uS7 was detected by anti-HA. TAP-Rio2 was detected by anti-CBP.

It was suggested that one of the docking targets of Rio2 in 43S ribosomes could be the C-terminal sequence of uS7 where authors showed direct interaction of these two proteins^16^. Recent progress in the field also suggested that cleavage of the D-site is achieved through the interaction of pre-43S ribosome with 60S ribosome to form pre-80S ribosome in the process leading to SSU maturation. In pre-80S ribosomes, 40S processing machinery digest at the D-site of 20S rRNA to give rise to mature 18S rRNA^28,29^. Nob1 is thought to be a nuclease in the protein complex^30^ and Rio2 is an essential ATPase bearing a protein kinase fold, presumably to create chemical force in the complex to digest rRNA^31^. It was suggested that an ATP-binding motif and neutral loop (N-loop) of Rio2 are responsible for pre-43S subunit-binding and subsequent release, respectively. Thus, mutation in the ATP-binding motif (*D253A*) causes compromised cellular proliferation in normal culture conditions, yet these cells are resistant to heat stress^32^ for unknown reasons. *RIO2-D253A* mutant cells attenuated cellular proliferation at 30 °C but were resistant at high temperature (37 °C). As reported^32^, a further mutation in N-loop, *RIO2-N-loop* rescued *RIO2-D253A* phenotype at 30 °C growth whereas heat stress resistance was retained as in the *RIO2-D253A* mutant (Fig. 6A). Although *S223A* mutation is toxic to *rio2*-mutants, S223A mutant cells survived better at 37 °C, indicating that *S223A* phenotype is similar to *RIO2-D253A* **(**Fig. 6A**)**. Genetic interaction of these genes suggested Rio2-uS7 interaction could be compromised by the *S223A* mutation. Apparent rescued phenotype of *rio2* mutations could be caused by the aberrant rRNA production found in both *rio2* and *S223A* mutants.

If the SSU maturation defect in the *S223A* mutant is caused by compromised Rio2-uS7 interaction, Rio2 should fail to co-participate in the 40S ribosome, thus this was experimentally evaluated. When sucrose gradient ultracentrifuge sedimentation samples were analyzed on Western blotting, uS7 was mainly observed with 40S and 80S ribosomes (Fig. 6B). As previously reported, a portion of Rio2 was co-sedimented with 40S ribosomes^12^. When quantified, about 41% of Rio2 was mainly detected in the fraction with 40S ribosomes in control cells with WT uS7 (Fig. 6C). In contrast, in *S223A* cells, Rio2 participation in 40S ribosomes was reduced to 28% and, in turn, a higher amount of Rio2 was detected in the top fractions which contain free-RNA species and cytosolic proteins (Fig. 6B,C). The interaction between Rio2 and uS7 was also detected in affinity purification, using a TAP-tagged Rio2 expressing strain. Here, S223A mutation reduces Rio2-uS7 interaction (Fig. 6D). These results were consistent with a previous report showing the uS7 C-terminal region to be involved in interaction with Nob1 and other maturation factors^16^. Therefore, Ser223 in the C-terminal region of uS7 could function as a key determinant of the interaction in the region. S223A mutant uS7 participated in the 40S fraction, indicating that S223A-containing 40S ribosomes could be formed. Notably, S223A also participated in 80S ribosomes (Fig. 5C), thus S223A-containing ribosomes could be further matured to 80S ribosomes. Since it is expected that abundance of mature 40S ribosomes is limited, S223A-containing 80S ribosomes could be pre-80s ribosomes, formation of which precedes 40S maturation, and accumulation is reported only in *rio1*-*D224A* overexpressed cells^29^. Collectively, these data suggested that *S223A* uS7 has a defect in the recruitment of ribosomal maturational protein Rio2 to 40S ribosomes, which could, in part, cause attenuated 40S ribosomes.

### Selective enhancement of heat shock protein translations in S223A mutant

**Figure 7:**
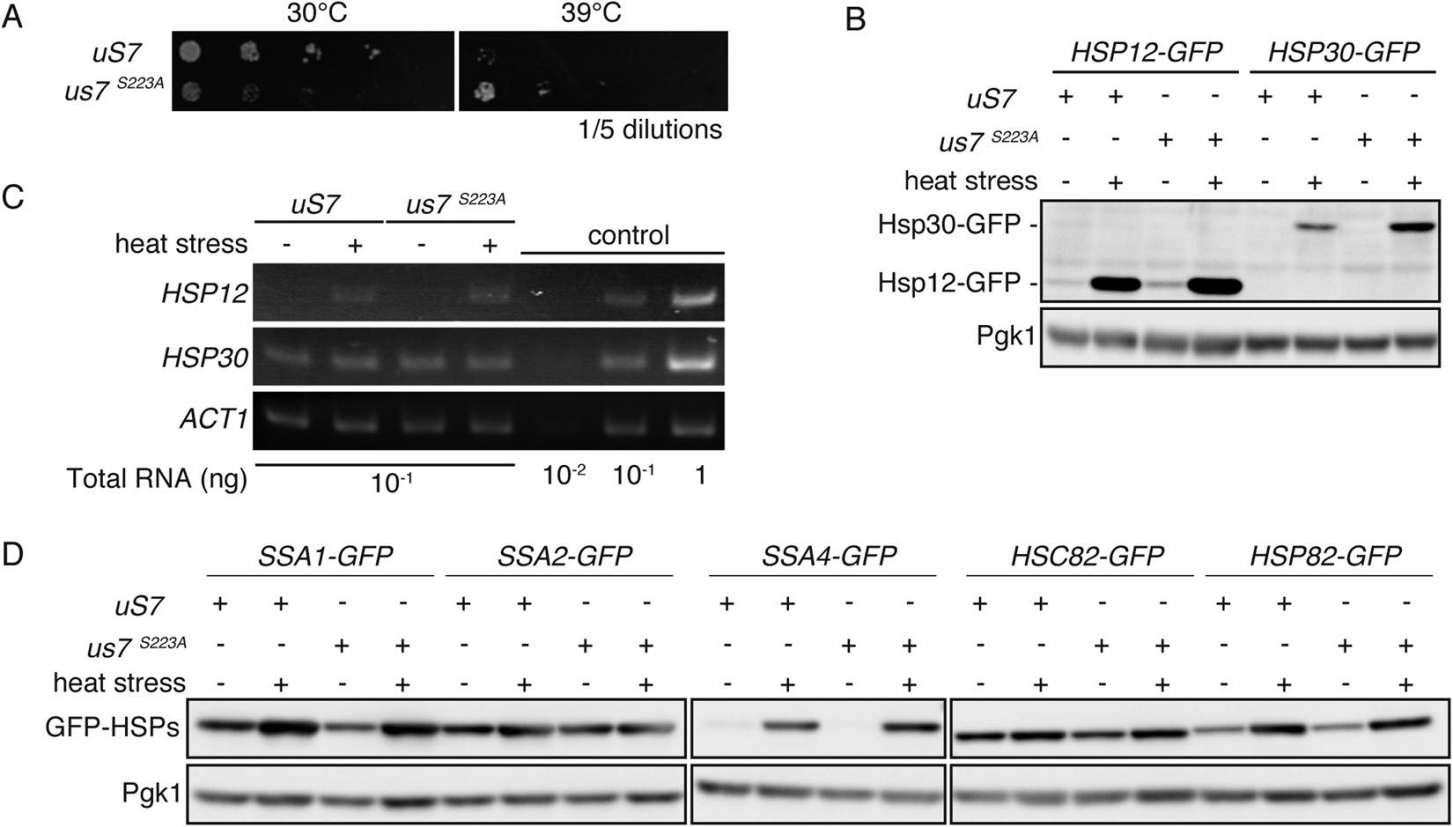
Enhanced heat stress resistance and enhanced translation of HSPs in *S223A* mutant. (**A**) Heat stress resistance of *S223A* mutant. Yeast cells were spotted on SD plate containing doxycycline as in Fig. 2D at different temperatures. Picture of plate was taken on day 2 for 30 °C and day 7 for 39 °C. (**B**) Enhanced heat shock protein expression in heat stressed cells in *S223A*. Induction of heat shock proteins was monitored utilizing GFP-knockin strains harboring indicated *uS7* plasmid. Heat stress was induced by placing culture flask in a water bath at 39 °C for 1 hr. After the exposure to heat stress, cells were harvested and lysed. Protein abundance was monitored by Western blotting with anti-GFP antibodies. Anti-Pgk1 was utilized as loading control. (**C**) Unaltered mRNA expression of heat shock proteins. Heat stressed cells were lysed and RNA was purified by hot-phenol method. Gene expression of *HSP12* and *HSP30* was quantified by RT-PCR experiments. In the conditions utilized, band intensities were dependent on the mRNA abundance. *ACT1* primers were used as loading control. (**D**) Enhanced specific heat shock proteins abundance in heat stressed *S223A* cells. Additional heat shock proteins were assessed as in Fig. 7B.

*S223A* mutation of uS7 could simply result in compromised ribosomes, deficient in translation ability. Given that S223A uS7 was still detectable with lower abundance compared to WT uS7, at least a minor population of ribosomes were properly assembled and functional in the *S223A* mutant. To explore a possible functional shift in S223A cells, heat stress resistance was analyzed. In contrast to compromised cellular proliferation under normal temperature 30 °C, the *S223A* mutant strain grew faster than the WT strain at 39 °C (Fig. 7A). To understand the molecular mechanism underlying heat resistance, heat shock protein (HSP) abundance was monitored. Abundance of Hsp12 and Hsp30 (of small HSP family) was elevated in the *S223A* cells (Fig. 7B), especially upon heat stress exposure at 39 °C for 1 hr. This increase was not due to levels of gene expression because mRNA levels examined in RT-PCR experiments were comparable (Fig. 7C). These data indicated that translation of these HSPs is augmented in *S223A* cells. Other HSPs were also examined. In WT cells, HSP70 family Ssa2 abundance was not elevated by heat shock while Ssa1 (HSP70 family), Hsc82 and Hsp82 (HSP90 family) were, however, the *S223A* mutation did not enhance the latter three proteins. Similar to Hsp12 and Hsp30, Ssa4 protein was detected exclusively in heat stressed cells and the *S223A* mutation further enhanced its abundance (Fig. 7D). Collectively, group-specific induction of HSPs such as Hsp12, Hsp30 and Ssa4 could be partly responsible for heat stress resistance of *S223A* cells. These data indicated that the S223A mutation could have a positive impact in translation for some genes. When overall translational inhibition and elevated heat shock protein translation is coordinated as in *S223A* cells, cells are expected to become more heat stress-resistant in the context of ER stress induction upon heat exposure^33^.

## Discussion

### Protein kinases phosphorylating uS7

Present data indicated that nutrient/stress-regulated protein kinases, Ypk1 and Pkc1 could regulate ribosomal maturation by phosphorylation of uS7 protein. Both Ypk1 and Pkc1 belong to the protein kinase family, AGC protein kinases, whose closest mammalian relatives are Akt, SGK and the PKC family^26,34^. Akt1 is involved in insulin signaling^35^ to control translation, therefore a phosphorylation event at uS7 could be conserved in mammalian systems. Interestingly, both Ypk1 and Pkc1 are phosphorylated in the activation loop by PDK1 homologs, Pkh1/2, in nutrient mediated signaling^36^. In addition, Ypk1 and Pkc1 are also regarded as downstream of TORC2, thus not only TORC1^37^, but also TORC2^11^ could be involved in cellular signaling phosphorylating ribosomal proteins. It is also notable that Pkc1 signaling is reported to control mRNA catabolism^36^, indicating that these kinases could be involved in various post-transcriptional steps to control cellular translation, depending on extracellular cues.

### Ypk1 in ribosomal protein phosphorylation

That Ypk1 could phosphorylate R-protein is interesting because Ypk1 was proposed to regulate translation in a TORC1-independent pathway^7^. Ypk1 proteolysis is controlled by an autophagy-related process under nitrogen starvation^38^, thus a Ypk1-mediated phosphorylation event could be stalled in nitrogen starved cells. Ypk1 could also phosphorylate LSU protein uL3 which has a consensus sequence for Ypk1-mediated phosphorylation^23^. It is possible that attenuation of both LSU and SSU in the *ypk1∆* strain could be caused by such a phosphorylation event.

### uS7 protein

The C-terminus of uS7 is important, and mutation phenotypes differ. Depletion phenotype of the *uS7* gene was classified as an “early defect” in ribosome biogenesis^13^. When mutant *us7* was used to rescue the deletion mutant, N-terminal deletion caused poor translational fidelity^15^. Likewise, C-terminus deletions caused “late defect” in 40S SSU maturation. Here, S223A mutant protein is localized in cytosol (Supplemental Fig. S7), indicating that early steps of ribogenesis in the nucleus was not affected, although expression of S223A protein caused a dominant-negative growth defect (Fig. 2C), as previously reported^19^. Other point mutations closer to this site did not show growth defects but exhibit start codon selection^18^. Therefore, multiple protein recognition events concerning this region could regulate protein translation. Due to its importance, it is not surprising that the C-terminal region is conserved throughout evolution from bacteria to mammals including yeast. In this sense, it is notable that plant uS7 has a natural S223A sequence (Supplemental Fig. S9). This indicates that plants seem to have a different regulatory system regarding uS7 C-terminal peptide modification.

### Heat shock protein translation in *S223A* cells

*S223A* cells effectively translated some HSPs such as Hsp12, Hsp30 and Ssa4 (Fig. 7), despite overall translation being attenuated (Fig. 3C) and mRNA levels were not different (Fig. 7C). This phenomenon cannot be explained unless overall translation and heat stress translation are independently regulated. It could be the case that preferential translation could be achieved by the ribosome species with a defect in Ser223 phosphorylation. Alternatively, lowering of the 40S ribosome level may be responsible for the selective translation in heat stressed cells, although molecular regulatory mechanism(s) involved in the enhanced translation of specific sets of genes under attenuated level of 40S ribosomes seems elusive at this moment. It was previously shown that mRNA species derived from promoters containing *heat shock element* (*HSE*) could preferentially escape from recruitment into stress granules and thus be preferentially translated upon heat stress^39^. Although *HSE* is a promoter element that functions to recruit transcriptional factors for transcription, this preference in the translation also utilizes the same *HSE*. Here, three HSPs exhibited stimulated protein abundance upon heat stress in *S223A* cells (Fig. 7). Among these three, Hsp12 and Hsp30 showed increased translation upon heat stress^39^. Therefore, an *S223A* mutation could potentiate *HSE*-mediated stress-induced translation. Such potentiated translation seems to be an effective regulation in the case of cells to cope with emergent environmental situations such as stress exposure. Difference in phosphorylation of uS7 could be an example for the regulation of much anticipated target-specific protein translation dependent on the *cis*-elements^20,40^. It is recently reported that uS7 and eIF2α could interact to recognize start codons^19^, thus S223A mutant and non-phosphorylated uS7 could favor translational initiation of “*HSE*”-controlled genes in stressed conditions.

### Monitoring Ser223 phosphorylation

Present results indicated that a phosphorylation event on uS7 Ser223 could be important for SSU maturation. It is not clear whether Ypk1-mediated phosphorylation on Ser223 is important for maturation. A difficulty to investigate this was that Ser223 phosphorylation could not be monitored, due to inefficient antigenicity of the phospho-peptide when antibody production was attempted. Phos-tag-mediated gel retardation assay did not work properly on this protein because an apparent phosphorylation-independent shift was found (data not shown). Nevertheless, monitoring the phosphorylation at this site is the next important step. Moreover, Ser223 phosphorylation could be controlled not only by Ypk1, but Pkc1. Probably due to the complex regulation by protein kinases, at least including Ypk1 and Pkc1, *ypk1∆* phenotype on ribosome regulation was not completely phenocopied with the *S223A* mutation. One important target for nutrient and stress signals is ribosomal biogenesis and translation^37^. Therefore, the present result could be characterized as an example of a novel phosphorylation event controlling translation, the most expensive cellular process downstream of the nutrient and stress signals.

## Methods

### Yeast strains and culture conditions

Yeast strains and plasmids used in this study are listed in Supplemental Table S2. Construction of deletion strains was achieved through PCR-based homologous recombination. Conditions for canonical cell culture and plating assays were as reported previously^41^. To induce transcription of *rDNA* from the *GAL7* promoter, cells were pre-cultured in SD medium containing 2% raffinose. The cells, grown until they reached to mid-log phase, were inoculated into SD medium containing 2% galactose and harvested at the indicated time.

### Plasmids and antibodies

Plasmids used in this study are listed in Supplemental Table S3. To construct the expression plasmid, *Saccharomyces cerevisiae* genes were amplified by PCR, and PCR products were ligated into each vector. All mutated expressing plasmids were generated by site-directed mutagenesis (Clontech Laboratories).

Polyclonal antibodies used against Ypk1^42^ and eL19 were raised in rabbit. The monoclonal antibody against Pgk1, HA, GFP, were obtained from Invitrogen, Covance and Santa Cruz Biotechnology, the latter which also supplied polyclonal antibodies against HA and Rio2. Polyclonal antibodies against CBP were purchased from Medical and Biological Laboratory (MBL). HRP-conjugated secondary antibodies against mouse IgG and rabbit IgG were obtained from Zymed Laboratories Inc.

### Identification of Ypk1 substrates by 2D-PAGE and mass spectrometry

For two-dimensional gel electrophoresis, *ypk1∆* cells or control yeast cells were harvested at OD_600_ = 0.6, which represents the mid-log phase. Cells were resuspended in 2D-PAGE lysis buffer [50 mM Tris-HCl pH 8.0, 150 mM NaCl, 60 mM DTT containing Roche complete protease inhibitor cocktail tablet and SIGMA Phosphatase inhibitor cocktails 1 and 2] and continuously vortexed for 30 min at 4 °C in a microtube mixer (model MT-360, Tomy, Tokyo, Japan) with a half-volume of glass beads to lyse the yeast cells. Unbroken cells and debris were removed by ultracentrifugation at 100000 × g for 60 min. Supernatant factions were treated with DNase and RNase to degrade nucleic acids and reductively alkylated before precipitated by cold acetone. Samples were dissolved in swelling buffer [7 M Urea, 2 M thiourea, 2% CHAPS, 2% Triton X-100, 100 mM DTT, 2 mM Tris(2-carboxyethyl)phosphine hydrochloride and 2.5 mM acetic acid]. Isoelectric focusing electrophoresis was performed on 11 cm ReadyStrip IPG Strips pH5-8 (Bio-Rad). The second electrophoresis was performed on 15% polyacrylamide gels by standard SDS-PAGE procedures. Gels were stained with ProQ-Diamond (Invitrogen), subsequently stained with SYPRO-Ruby (Invitrogen) and fluorescent images of gels were acquired. Spots of interest on ProQ-Diamond/SYPRO-Ruby staining were subjected to in-gel digestion with 12.5 ng/mL trypsin and the resulting mixture analyzed by LC-MS/MS using an LTQ instrument (Thermo Electron). Data was analyzed using Mascot database-searching software (Matrix Science) (Perkins *et al*., 1999), which identifies proteins by matching mass spectrometric data with information held in the NCBI (http://www.ncbi.nlm.nih.gov) and Swiss-Prot (http://us.expasy.org) protein databases. Protein spots differentially stained with these dyes were identified and are listed in Supplemental Table S1.

### Western blotting

For normal one-dimensional gel electrophoresis, harvested yeast cells were resuspended in lysis buffer [50 mM Tris-HCl (pH7.6), 0.5 mM EDTA, 150 mM NaCl, 50 mM sodium fluoride, 30 mM β-glycerophosphate, 1 mM PMSF, protease inhibitor cocktail for general use (Nacalai Tesque), and 0.5% Triton X-100]. The cell suspension was continuously vortexed for 10 min at 4 °C in a microtube mixer (MT-360, Tomy) with a half-volume of glass beads to lyse the yeast cells. Unbroken cells and debris were removed by centrifugation at 500 × g for 10 min, and the supernatant fraction containing total 5 μg of protein was used for SDS-PAGE analysis. After separation by SDS-PAGE, proteins were transferred to nitrocellulose membranes and proteins were detected using antibodies. To detect bound antibodies, chemiluminescent substrates Chemilumi-One (Nacalai Tesque) or SuperSignal West Femto Maximum Sensitivity Substrate (Pierce) were used in conjunction with LAS-3000/4000 imager (Fujifilm, Tokyo, Japan).

For co-immunoprecipitation, cell lysates were pre-cleared by 1 hr incubation with Protein G Sepharose 4B or IgG Sepharose. Pre-cleared lysate was subsequently incubated with anti-Ypk1, anti-CBP or anti-HA polyclonal Abs. Washed protein-bound beads were eluted by boiling and analyzed by above-mentioned procedures.

### *In vitro* kinase assay

Recombinant GST-uS7, and GST-uS7^S223A^ were expressed in soluBL21 *Escherichia Coli* containing pGEX4T-1 plasmids and purified through binding to a glutathione-Sepharose 4B column and eluted by glutathione, as described previously^42^. The yeast cells overexpressing Ypk1, Ypk1^K376A^, HA-Ypk2 or Pkc1^R398P^ were lysed and immunoprecipitated using protein G Sepharose and anti-Ypk1, anti-HA or anti-Pkc1 antibodies. In some experiments, pGAL-*GST-YPK1* lysate was used for enzyme source and purified with glutathione-Sepharose resin. The immune precipitants were used for enzyme source, thus resuspended in kinase reaction buffer [50 mM Tris-HCl pH 7.5, 200 mM NaCl, 10 mM MgCl_2_, 0.1 mM EDTA 10 μM ATP] with substrates recombinant uS7 prepared from *E. coli* and [γ-^32^P]-ATP (NEG-502Z, Perkin Elmer). Reaction mixture was incubated for 30 min at 30 °C with tapping every 3 min and reaction was terminated by the addition of 5x SDS-PAGE sample buffer. Samples were heated at 98 °C for 5 min. Proteins were separated by SDS-PAGE and the gels were subsequently stained with CBB and dried. Phosphorylated proteins were detected by autoradiography using BAS-2500 imaging system (Fujifilm). Due to the presence of the phosphorylation signal from Sepharose-precipitate unspecified kinase, Ypk1-mediated phosphorylation was monitored as an increment from the control, in which same Sepharose resin was used from *ypk1∆* lysate.

### *De novo* translational analysis

For ^35^S metabolic labeling, log-phase cells (total OD_600_ = 3.0) were washed with methionine-free SD medium to temporary stall translation and metabolically labeled at 30 °C with 100 μCi [^35^S]-methionine/cysteine (EXPRE^35^S^35^S protein labeling mix, Perkin Elmer)-containing SD medium to restore the translation. After incubation, metabolic labeling was terminated by washing cells with medium.

For analysis of radioactive amino acid uptake activity of strains, metabolic labeling was terminated with indicated times and a small aliquot of the washed yeast (total OD_600_ = 0.03) was directly lysed in scintillation cocktail and radioactivity was measured in the ^35^S-channel with Tri-Carb Liquid Scintillation Analyzer (Perkin Elmer) to quantify cellular [^35^S]-amino acids uptake of each strain.

Cellular translation activity was quantified as [^35^S]-incorporation into *de novo* translation. Briefly, cells were incubated at 30 °C with labeling mix for 10 minutes to allow *de novo* protein biosynthesis. After incubation, cells were washed with SD medium and lysed in lysis buffer [50 mM Tris-HCl, 0.5 mM EDTA, 150 mM NaCl, 50 mM sodium fluoride, 30 mM β-glycerophosphate, 1 mM PMSF, protease inhibitor cocktail for general use (Nacalai Tesque), and 0.5% Triton X-100]. Cellular proteins were recovered with TCA precipitation. Protein concentration in the precipitate was measured by the Bradford method and radioactivity was measured with liquid scintillation counting. Translation activity of each strain was expressed as radioactivity per protein content (dpm/μg protein). To normalize three independent experiments, this translation activity value was normalized as a ratio to that of WT strain in each experiment and the mean ratio was plotted. To rule out the possibility that protein could be degraded in mutant cells, a TCA precipitate was also analyzed with SDS-PAGE and equal CBB staining and attenuated radioactive protein abundance was confirmed in one of the triplicated experiments. In such experiment, radioactivity of the whole lane was analyzed by the use of BAS2500 (Fuji Film) radioactive image reader.

### Sucrose gradient fractionation of ribosomes

Polysome analyses were carried out as previously reported^27^, briefly, 6 × 10^9^ yeast cells were lysed in TSM lysis buffer (10 mM Tris-HCl pH7.4, 100 mM NaCl, 30 mM MgCl_2_ in DEPC water) with glass beads, and cleared lysate containing 900 μg total RNA was subjected to centrifugation with a 10–40% sucrose gradient. Gradient Station model 153 (BioComp Instrument, Inc.) was used to make the sucrose gradient and also to fractionate the post-ultracentrifuge samples. A_260_ was measured for RNA and peaks were assigned to each ribosomal species examining the presence of uS7 (SSU) and eL19 (LSU) ribosomal proteins in each fraction.

### Flow cytometry

To analyze the cell cycle, mid-log phase yeast cells were synchronized with addition of 10 μg/mL α factor and arrested in G_1_ phase for 3 hr at 30 °C. G_1_-arrested cells were washed with normal SD medium for cell cycle progression. Cells were harvested at indicated time points and fixed with 70% ethanol overnight. Cellular RNA was digested with 0.25 mg/mL RNase A treatment. Chromosomal DNA was stained with 50 μg/mL propidium iodide and analyzed by FACSCalibur (Becton, Dickinson).

### RNA purification and analysis

To extract total RNA from yeast cells, the MasterPure Yeast RNA Purification Kit (Epicentre Biotechnologies) was used. Northern blot hybridization was performed as previously described^27^. Oligo DNA sequences used for probes are listed in Supplemental Table S4.

To quantify rRNA abundance, qRT-PCR was performed utilizing the SuperScript III Platinum SYBR Green One-Step qRT-PCR Kit with ROX (Invitrogen) with Applied Biosystems 7500 Real-time PCR System by the standard curve method. The semi-quantitative RT-PCR was performed as described previously^38^.

### Northern Blotting

RNA pol II-dependent ribosomal RNA transcription and maturation was monitored in Northern blotting. Ribosomal DNA mini gene with *GAL7* promoter and tagged sequence was utilized in the experiments. Yeast cells were pre-cultured in raffinose-containing medium and ribosomal RNA transcription was driven after switching to galactose-containing media at density of OD_600_ = 0.5. After indicated time, cells were harvested and total RNA was purified. RNA samples (1.6 μg) were analyzed with Northern blotting utilizing 5’ radio-labeled tagged oligonucleotide probes specific for 18S and 25S rRNA, which could detect only mini gene-derived rRNA species. To compare rRNA abundance of *uS7* and *S223A* cells, samples were loaded and analyzed side-by-side within the same membrane. Radioactivities of blotting bands were quantified using BAS2500 radioactivity imager exposure. Here, 100% value corresponds to maximum band signal in each blot for either 18S or 25S probe. Triplicate experiments were performed and mean relative value was plotted with SD-error.

## Electronic supplementary material

Supplemental Information
