# Supplementary material for: Ribosomal protein uS7/Rps5 serine-223 in protein kinase-mediated phosphorylation and ribosomal small subunit maturation
Source: Sci Rep. 2018 Jan 19;8:1244. doi: 10.1038/s41598-018-19652-z (PMC5775349; doi:10.1038/s41598-018-19652-z)

Supplemental text/tables/figures/images for

Title

Ribosomal protein uS7/Rps5 serine-223 in protein kinase-mediated phosphorylation and ribosomal small subunit maturation

Authors

Makoto Tomioka [a, b], Mitsugu Shimobayashi [b, †], Makoto Kitabatake [c], Mutsuhito Ohno [c], Yasunori Kozutsumi [b], Shogo Oka [a], Hiromu Takematsu [a, b] *

Supplemental Table S1

Screening of proteins affected in *ypk1*Δ strain.

Logarithmically growing yeast cells were harvested at OD_{600}=0.6 and cytosolic protein fractions were prepared. Samples were first separated with nonequlibrium pH gel electrophoresis and then SDS-PAGE. Gels were then stained with SYPRO Ruby and Pro-Q Diamond reagents to visualize levels of protein abundance and phosphorylation, respectively. Thirteen different spots picked up for mass spectrometry analysis from 2D gels resulted in candidate peptide identification. This table shows the candidate molecules responsible for each spot. Increase and decrease of abundance was indicated as "+" and "-", respectively. Spot ID 16 further confirmed that uS7 is the protein responsible by the following experiments in this manuscript.

Spot ID	Std name	Systematic name	protein abundance	phosphorylation
C	Hxk1	Yfr053c		+
F	Rbi1	Ydl135c		+
2	Adi1	Ymr009w	+	
3	Tpi1	Ydr050c	-	-
3	Rib3	Ydr487c	-	-
7	Tdh2	Yjr009c	-	
8	Ald6	Ypl061w	-	
8	Mrt4	Ykl009w	-	
9	Hyp2	Yel034w	-	
10	Eno1	Ygr254w	+	
11	Pdc1	Ylr044c	+	
16	uS7/Rps5	Yjr23w	-	-
17	Rpp2b	Ydr282w	+	+
17	Efb1	Yal003w	+	+
19	Tdh3	Ygr192c	+	
28	Pst2	Ydr032c	+	+

Supplemental Table S2

Yeast strains used in this study.

Strain	Genotype	Source / Reference
SEY6210	*MATa leu2-3.112 ura3-52 his3-Δ200 trp1-Δ901 lys2-801*	(Robinson et al., 1988)
ypk1Δ(6210)	SEY6210 *ypk1*::*KanMX6*	(Tanoue et al., 2005)
BY4741	*MATa his3-1 leu2-0 met15-0 ura3-0*	(Brachman et al., 1998)
ypk1Δ(BY)	BY4741 *ypk1*::*HISMX6*	(Shimobayashi et al., 2010)
ypk2Δ	BY4741 *ypk2*::*URA3*	This study
HA-uS7	BY4741 *puS7::KanMX4-pRPS5-HA*	This study
ypk1Δ/HA-uS7	BY4741 *ypk1*::*HISMX6 puS7::KanMX4-puS7-HA*	This study
R1158	BY4741 *ura3-0*::*CMV-tTA*	Open biosystems
ypk1Δ(R1158)	R1158 *ypk1*::*HISMX6*	This study
pTetoff-uS7	R1158 *puS7*::*KanMX4-TetO7CYCTATApuS7*	Open biosystems
ypk1Δ/pTetoff-uS7	R1158 *puS7::KanMX4-TetO7CYCTATApuS7 ypk1::HISMX6*	This study
HSP12-GFP	BY4741 *HSP12::HSP12-GFP-HISMX6*	Invitrogen
HSP30-GFP	BY4741 *HSP30::HSP30-GFP-HISMX6*	Invitrogen
SSA1-GFP	BY4741 *SSA1::SSA1-GFP-HISMX6*	Invitrogen
SSA2-GFP	BY4741 *SSA2::SSA2-GFP-HISMX6*	Invitrogen
SSA4-GFP	BY4741 *SSA4::SSA4-GFP-HISMX6*	Invitrogen
HSC82-GFP	BY4741 *HSC82::HSC82-GFP-HISMX6*	Invitrogen
HSP82-GFP	BY4741 *HSP82::HSP82-GFP-HISMX6*	Invitrogen
RIO2-TAP	BY4741 *RIO2::RIO2-TAP-HISMX6*	Dharmacon

Supplemental Table S3

Plasmid constructs used in this study

Plasmid name	Features	Reference
pRS413	*CEN; HIS3*	NEB
pRS413-uS7	*CEN; HIS3; uS7* ORF under own promoter	this study
pRS413-us7 S223A	*CEN; HIS3; uS7* mutant ORF under own promoter	this study
pRS413-2xHA-uS7	*CEN; HIS3; uS7* ORF fused to 2xHA tag	this study
pRS413-2xHA-us7 S223A	*CEN; HIS3; uS7* mutant ORF fused to 2xHA tag under own promoter	this study
pRS415	*CEN; LEU2*	NEB
pRS415-uS7	*CEN; LEU2; uS7* ORF under own promoter	this study
pRS415-us7 S2A, T4A	*CEN; LEU2; uS7* mutant ORF under own promoter	this study
pRS415-us7 T21A, T27A	*CEN; LEU2; uS7* mutant ORF under own promoter	this study
pRS415-us7 S57A	*CEN; LEU2; uS7* mutant ORF under own promoter	this study
pRS415-us7 T73A	*CEN; LEU2; uS7* mutant ORF under own promoter	this study
pRS415-us7 T146A, T147A	*CEN; LEU2; uS7* mutant ORF under own promoter	this study
pRS415-us7 T189A	*CEN; LEU2; uS7* mutant ORF under own promoter	this study
pRS415-us7 S223A	*CEN; LEU2; uS7* mutant ORF under own promoter	this study
pRS415-us7 S223D	*CEN; LEU2; uS7* mutant ORF under own promoter	this study
pRS415-2xHA-uS7	*CEN; LEU2; uS7* ORF fused to 2xHA tag under own promoter	this study
pRS415-2xHA-us7 S223A	*CEN; LEU2; uS7* mutant ORF fused to 2xHA tag under own promoter	this study
pRS415-GFP-uS7	*CEN; LEU2; uS7* ORF fused to GFP tag under own promoter	this study
pRS415-GFP-us7 S223A	*CEN; LEU2; uS7* mutant ORF fused to GFP tag under own promoter	this study
pRS415-uS3-GFP	*CEN; LEU2; uS3* fused to the *GFP* under own promoter	this study
pRS415-uL23-GFP	*CEN; LEU2; uL23* fused to the *GFP* under own promoter	this study
pRS416	*CEN; URA3*	NEB
YEp351-YPK1	*2µ; LEU2; YPK1* ORF under own promoter	(Sun et al., 2000)
YEp351-YPK1(KD)	*2µ; LEU2;* $ypk1^{K376A}$ mutant ORF under own promoter	(Sun et al., 2000)
pGAL1(pEG(KG))	*2µ; URA3; GAL1* promoter vector	(Zhu et al., 2000) *
pGAL1-GST-YPK1	*2µ; URA3; YPK1* ORF fused to *GST* under *GAL1* promoter	(Zhu et al., 2000)*
YEp351-HA-YPK2	*2µ; LEU2; YPK2* ORF fused to 1xHA tag under own promoter	this study
YEp351-PKC1(CA)	*2µ; LEU2;* constitutively-active *PKC1* ORF under own promoter	(Inagaki et al., 1999) **
YEp351-RIO2-His6	*2µ; LEU2; RIO2* ORF fused to the His6 tag under *ADH1* promoter	this study

YEp351-rio2253A-His6	*2µ; LEU2; RIO2* mutant ORF fused to the His6 tag under *ADH1* promoter	this study
YEp351-rio2N-loop-His6	*2µ; LEU2; RIO2* mutant ORF fused to the His6 tag under *ADH1* promoter	this study
YEp351-rio2253A/N-loop-His6	*2µ; LEU2; RIO2* mutant ORF fused to the His6 tag under *ADH1* promoter	this study
pWT4-LEU2	*2µ; LEU2; rDNA 25S-tag* and *18S-tag* under *GAL7* promoter	this study
pGEX4T-1-uS7	E. coli expression; Amp; *uS7* ORF fused to the GST	this study
pGEX4T-1-uS7(S223A)	E. coli expression; Amp; *uS7* mutant ORF fused to the GST	this study

Kindly provided by Dr. Jason Ptacek*, Dr. Maiko Inagaki**.

Supplemental Table S4

Oligonucleotide sequence of probes and primers used in this study

Name	Sequence	Note	Used in
HSP12-Fwd	5'-AAGGATTCGGTGAAAAAGCTTCTGA	*HSP12* ORF sequence	sqRT-PCR
HSP12-Rev	5'-TGGGTCTTCTTCACCGTGGACACGA	complementary to *HSP12* ORF	sqRT-PCR
HSP30-Fwd	5'-ATATGCCTTAGCTCCTGCATTTTTG	*HSP30* ORF sequence	sqRT-PCR
HSP30-Rev	5'-TACCCACGATTTGAATTAACAGCGA	complementary to *HSP30* ORF	sqRT-PCR
ACT1-Fwd	5'-AGGTTGCTGCTTTGGTTATTGATAA	*ACT1* ORF sequence	sqRT-PCR
ACT1-Rev	5'-AACAGGGTGTTCTTCTGGGGCAACT	complementary to *ACT1* ORF	sqRT-PCR
kota125	5'-TACAGTAAACTGCGAATGGC	untagged 18S rRNA sequence	realtimePCR
kota152	5'-ATCTCTTCCAAAGGGTCGAG	complementary to untagged 18S rRNA	realtimePCR
kota030	5'-GAAATCTGGTACCTTCGGTG	untagged 25S rRNA sequence	realtimePCR
kota031	5'-GATTCTCACCCTCTATGACG	complementary to untagged 25S rRNA	realtimePCR
MK923	5'-CAGAAATCTCTCACCGTTTGG	complementary to untagged *ITS-1*	realtimePCR
MK924	5'-GCTTTTACTGGGCAAGAAGAC	untagged *ITS-1* sequence	realtimePCR
MK253	5'-CACCGAAGGTACACTCGAGA GCTTC	complementary to pWT4 25S rRNA tag	Northen blotting
kota153	5'-CGAGGATTCAGGCTTTGG	complementary to pWT4 18S tag	Northen blotting

Legends for Supplemental Figures

Supplemental Figure S1. Proteomic identification protein affected in *ypk1*Δ strain
Cytosolic proteins extracted from WT and *ypk1*Δ strain were separated by 2D-gel. Protein spots were visualized with SYPRO Ruby (for protein abundance) and Pro-Q Diamond (for protein phosphorylation). From each strain, corresponding pI value and molecular weight was indicated. Magnified image (boxed) is available in **Fig 1**.

Supplemental Figure S2. Structure of yeast ribosome complex
uS7 localization within the head of the yeast 80S ribosome complex. PDB file 4V88 was used for modeling. Image was produced using PyMOL software. The small subunit proteins are depicted in blue, and the large subunit proteins in green. uS7 was shown in yellow and its 223th Ser (arrow) was highlighted in red.

Supplemental Figure S3. Kinase activity toward uS7 in kinase assay
A. Ypk1 activity toward uS7.
Protein kinase activity of WT Ypk1, immunoprecipitated with anti-Ypk1 and protein G-Sepharose, toward uS7 was measured with kinase reaction buffer in the presence of [γ ^{32}P]-ATP. BAS-2500 radioactive imager (Fuji film) was used to measure ^{32}P incorporation after SDS-PAGE gel was CBB stained and dried. Presence of Ypk1 and uS7 was confirmed with Western blotting and CBB staining, respectively. Although presence of radioactive signals was detectable in the controls without anti-Ypk1 (left three lanes), enhancement of radioactivity was found with Ypk1 (right three lanes). Only a mild increase was found with incremental uS7 addition to the assay, indicating that Ypk1 activity was detected at a rate similar to the Vmax. Without addition of immunoprecipitated material, no radioactivity was found in the size of uS7, indicating that uS7 fraction does not have protein kinase activity toward uS7.
B. Ypk1 activity with glutathione-Sepharose precipitation.
To understand the radioactivity found in the control condition, different conditions of Ypk1 immunoprecipitation were tried. Control WT strain without *GST*-tagged *YPK1* vector was GST-precipitated with glutathione-Sepharose. Radioactivity was measured

as in A. Consistent background radioactivity was detected. Again, additive effect was observed in the condition with *GST-YPK1* plasmid. Therefore, this assay background is caused by the co-precipitated kinase protein with Sepharose resin. Ypk1 activity could be measured as additive effect from the background. GST-uS7 protein used as substrate was not stable more than a week, thus GST-uS7 protein was freshly prepared for each kinase assay. Apparent signal strength varied by the [γ ^{32}P]-ATP, thus this reagent was used within one half-life of ^{32}P.

Supplemental Figure S4. Phospho-mimetic mutation

Alanine, aspartate or glutamate mutation was introduced to uS7-S223 site. Mutant uS7s were expressed from plasmid vector pRS415 with own promoter in the condition endogenous uS7 was turned off utilizing Tet-OFF system with 10 μg/mL doxycycline. 5-fold serial dilutions of indicated yeast cells were spotted onto SD plate and incubated at 30˚C as in **Fig 2C**.

Supplemental Figure S5. Cellular uptake of radiolabeled methionine-cysteine

Yeast cells (3.0 OD) of indicated genotypes were incubated with 100 μCi of [^{35}S] Met-Cys after methionine depletion. After harvesting cells at indicated time points, cells were washed and cellular amino acid uptake efficiency was monitored by radioactivity measurement with liquid scintillation counting. Data was expressed as radioactive uptake per given cell number (dpm/OD).

Supplemental Figure S6. Probes used for qPCR for ribosomal maturation

Location of the primers used in the PCR experiments was depicted. Actual primer sequences are available in **Supplemental Table S4**.

Supplemental Figure S7. Subcellular localization of uS7

Logarithmically growing yeast cells was harvested and GFP-fluorescence was visualized by confocal microscopy. Result from *GFP-uS7* and *GFP-S223A* expressing strain is shown. Bars indicate 10 μm. Unchanged subcellular localization but attenuated signal strength was detected in the *GFP-S223A* expressing strain.

Supplemental Figure S8. Polysome assay of *ypk1*Δ strain

Logarithmically growing WT and *ypk1*Δ cells were analyzed for ribosomes in the sucrose gradient ultracentrifugation assay as in Fig5 C, D. Ribosome profile of these two samples were similar probably due to the compensation in the *ypk1*Δ cells, which exhibited reduction of both SSU and LSU. Consequently, subtle increase in the polysome population was found in *ypk1*Δ cells.

Supplemental Figure S9. Evolutional conservation of uS7 C-terminal sequences

Amino acid sequence alignment of uS7 from *D. melanogaster*, *H. sapiens*, *C. elegans*, *C. reinhardtii*, *A. thaliana*, and *S. cerevisiae*. Image was produced with CLUSTAL 2.1. Identical and similar amino acid residues were marked. Arrow indicates the position, Ser 223.

Supplemental Gel Images 1.

When blotting results were clipped from original whole images in manuscript figures to save the space, whole images were presented as supplemental gel images. All of full-length or whole blot images (for those when blot was cut for multiple blotting probes) used for the Figures 1, 2 and 4 were shown as indicated. Clipped images in main figures were indicated as dotted boxes.

Supplemental Gel Images 2.

When blotting results were clipped from original whole images in manuscript figures to save the space, whole images were presented as supplemental gel images. All of full-length or whole blot images (for those when blot was cut for multiple blotting probes) used for the Figures 5, 6 and 7 were shown as indicated. Clipped images in main figures are indicated as dotted boxes.

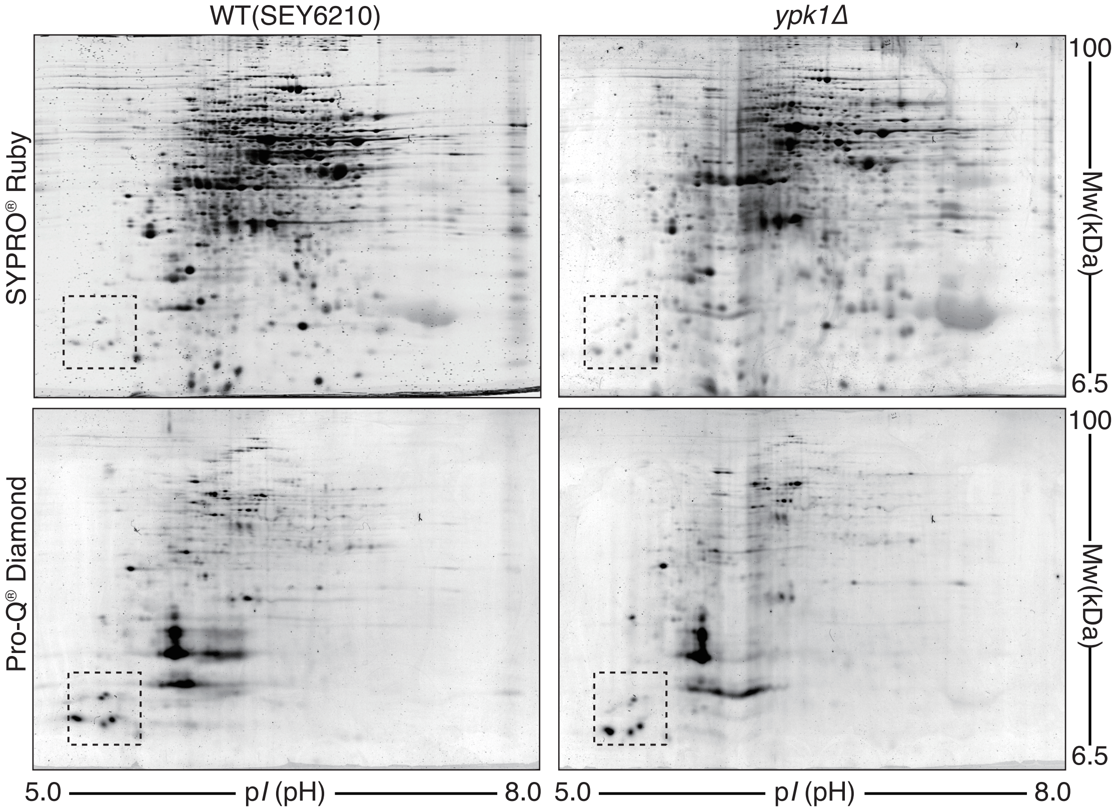

WT(SEY6210)
ypk1Δ
SYPRO® Ruby
Pro-Q® Diamond
100
Mw(kDa)
6.5
5.0
pI (pH)
8.0

Supplemental Figure S2

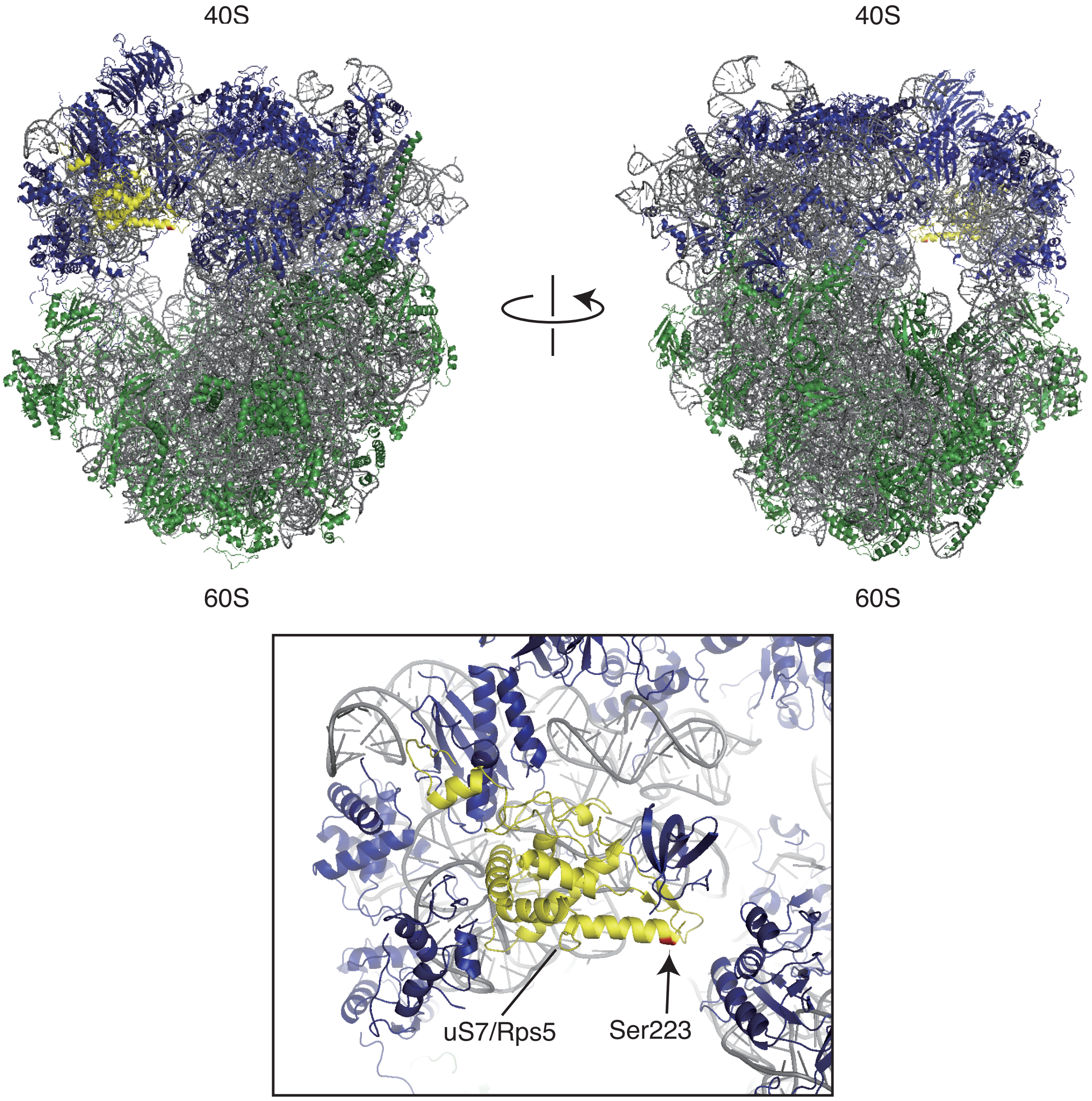

Supplemental Figure S3

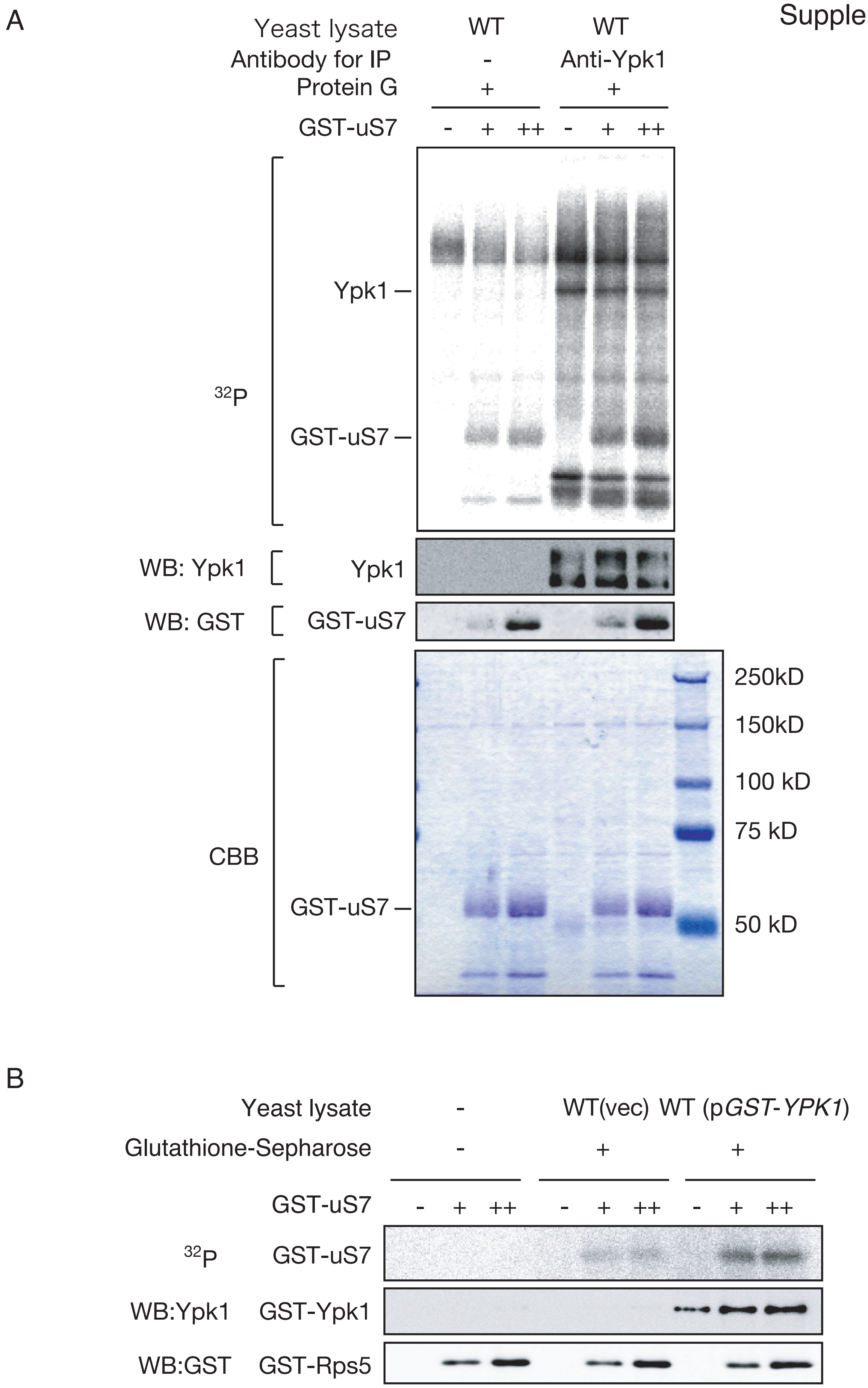

Supplemental Figure S4

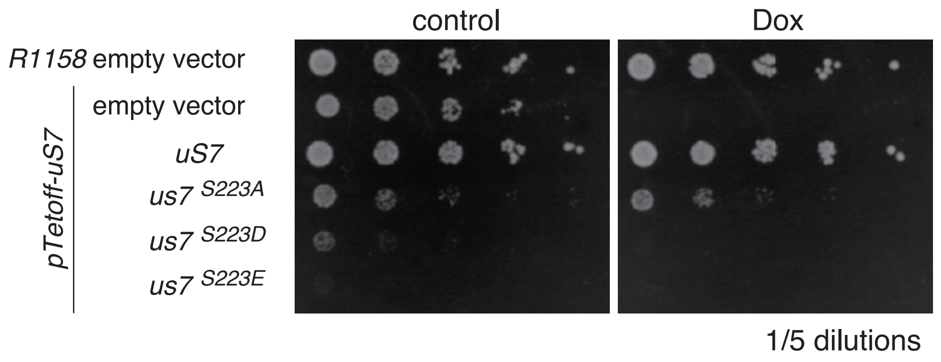

Supplemental Figure S5

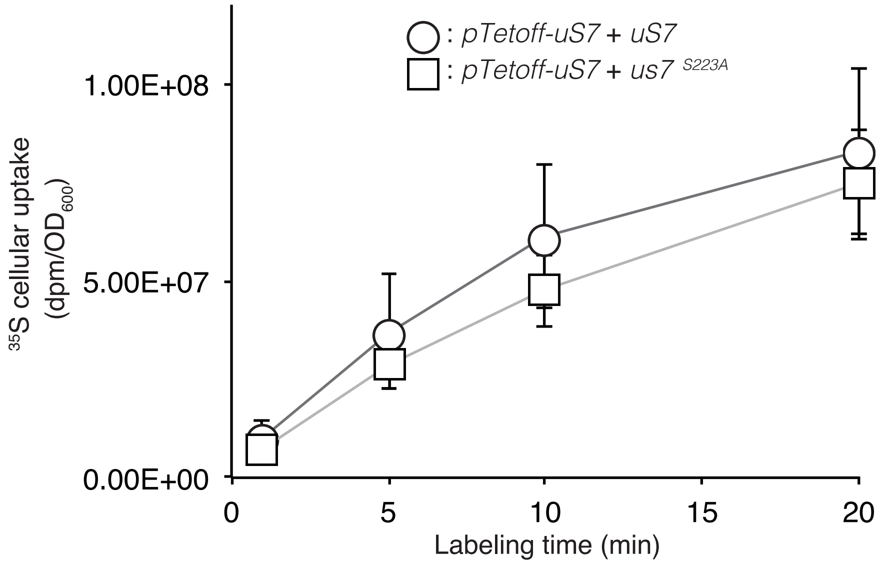

Supplemental Figure S6

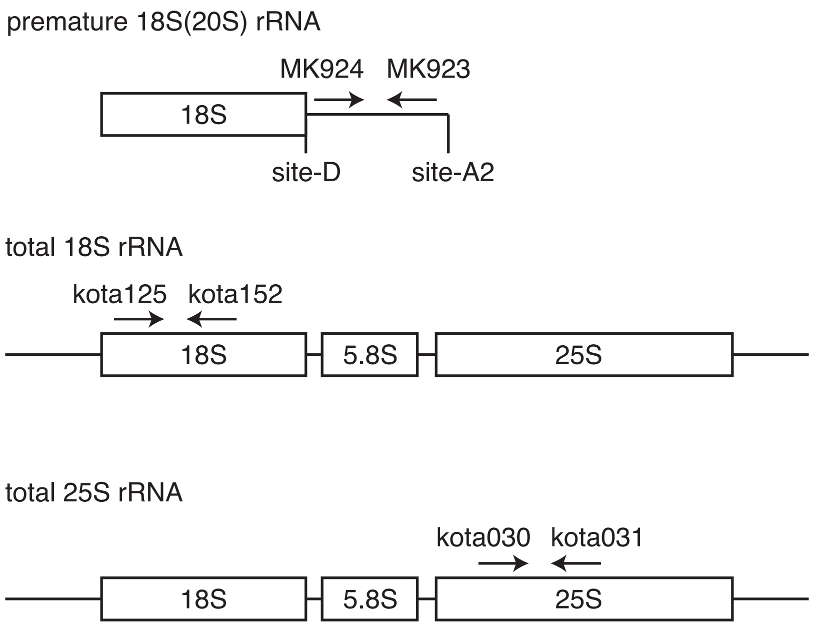

Supplemental Figure S7

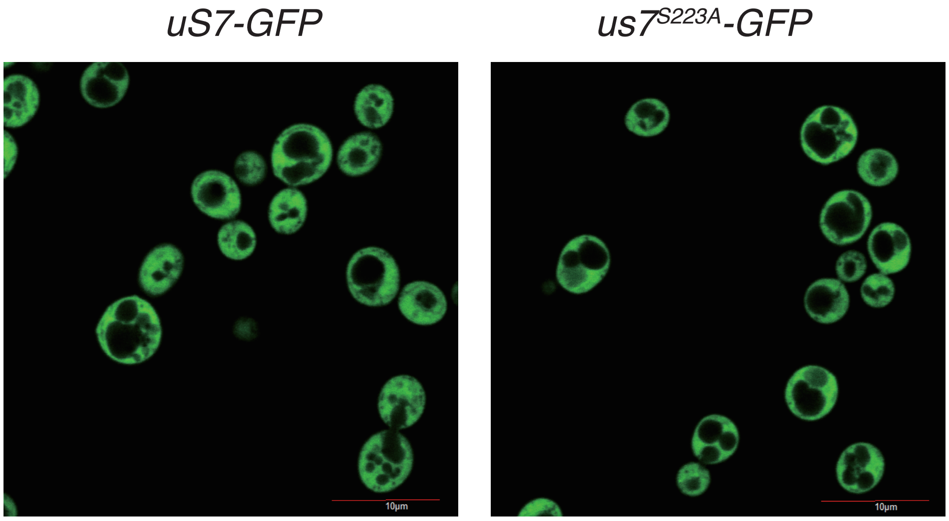

Supplemental Figure S8

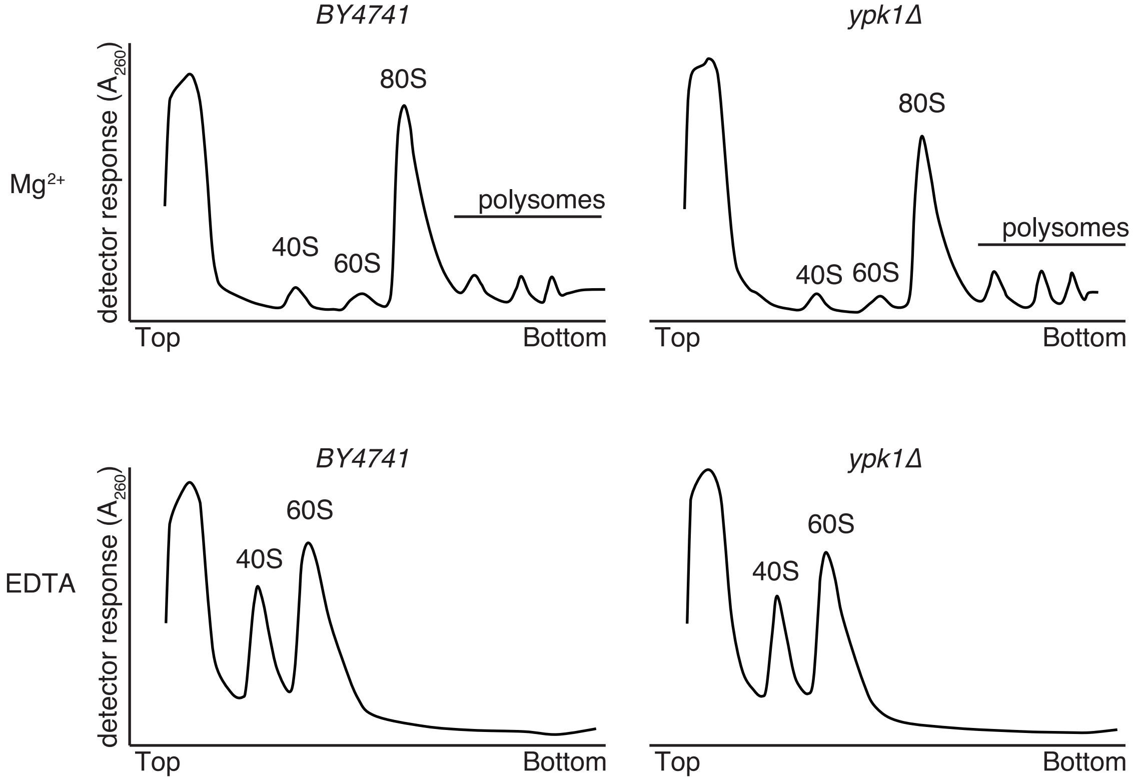

Supplemental Figure S9

```
CLUSTAL 2.1 multiple sequence alignment

D.melanogaster      MAEVAENVVETFEEPAAPMEAEVAETILETNVVSTTELPEIKLFGRWSCDDVTVNDISLQ 60
H.sapiens           ------------------------MTEWETAAPAVAETPDIKLFGKWSTDDVQINDISLQ 36
C.elegans           ------------------MADNWGSENVVADAAPATEAPEVALFGKWSLQSVNVSDISLV 42
C.reinhardtii       -----------------------------------MASPDVKLFGKWSFEDIEVTDISLE 25
A.thaliana          ----------------------MATAADVDAEIQQALTNEVKLFNRWTYDDVTVTDISLV 38
S.cerevisiae        MSDTEAPVEVQEDFEVVEEFTPVVLATPIPEEVQQAQT-EIKLFNKWSFEEVEVKDASLV 59
                                                           :: **.:*: :.: :.* **

D.melanogaster      DYISVKE-KFARYLPHSAGRYAAKRFRKAQCPIVERLTCSLMMKGRNNGKKLMACRIVKH 119
H.sapiens           DYIAVKE-KYAKYLPHSAGRYAANAFRKAQCPIVERLTNSMMMHGRNNGKKLMTVRIVKH 95
C.elegans           DYIPVKE-KSAKYLPHSAGRFQVRRFRKAACPIVERLANSLMMHGRNNGKKLMTVRIVKH 101
C.reinhardtii       DYIAVKT-KYAVYVPHTAGRYQKRRFRKALCPIVERLCNSLMMHGRNNGKKLMAVRIVKH 84
A.thaliana          DYIGVQAAKHATFVPHTAGRYSVKRFRKAQCPIVERLTNSLMMHGRNNGKKLMAVRIVKH 98
S.cerevisiae        DYVQVRQ---PIFVAHTAGRYANKRFRKAQCPIIERLTNSLMMNGRNNGKKLKAVRIIKH 116
                    **: *:    . ::.*:***:  . **** ***:***  *:**:******** : **:**

D.melanogaster      SFEIIHLLTGENPLQILVSAIINSGPREDSTRIGRAGTVRRQAVDVSPLRRVNQAIWLLC 179
H.sapiens           AFEIIHLLTGENPLQVLVNAIINSGPREDSTRIGRAGTVRRQAVDVSPLRRVNQAIWLLC 155
C.elegans           AFEIIYLLTGENPVQVLVNAVINSGPREDSTRIGRAGTVRRQAVDVAPLRRVNQAIWLLC 161
C.reinhardtii       AFDIIHLLTDQNPIQVVVDAIINSGPREDATRIGSAGVVRRQAVDISPLRRVNQAIYLLT 144
A.thaliana          AMEIIHLLSDLNPIQVIIDAIVNSGPREDATRIGSAGVVRRQAVDISPLRRVNQAIFLIT 158
S.cerevisiae        TLDIINVLTDQNPIQVVVDAITNTGPREDTTRVGGGGAARRQAVDVSPLRRVNQAIALLT 176
                    :::** :*:. **:*:::.*: *:*****:**:* .*..******::********* *:

D.melanogaster      TGAREAAFRNIKTIAECLADELINAAKGSSNSYAIKKKDELERVAKSNR 228
H.sapiens           TGAREAAFRNIKTIAECLADELINAAKGSSNSYAIKKKDELERVAKSNR 204
C.elegans           TGAREAAFRNVKTIAECLADELINAAKGSSNSYAIKKKDELERVAKSNR 210
C.reinhardtii       TGAREAAFRNIKTIAECLADELVNAAKGSSNRYAIKKKDEIERVAKANR 193
A.thaliana          TGAREAAFRNIKTIAECLADELINAAKGSSNSYAIKKKDEIERVAKANR 207
S.cerevisiae        IGAREAAFRNIKTIAETLAEELINAAKGSSTSYAIKKKDELERVAKSNR 225
                     *********:***** **:**:*******. ********:*****:**
```

Supplemental Gel Images 1

Figure 1B

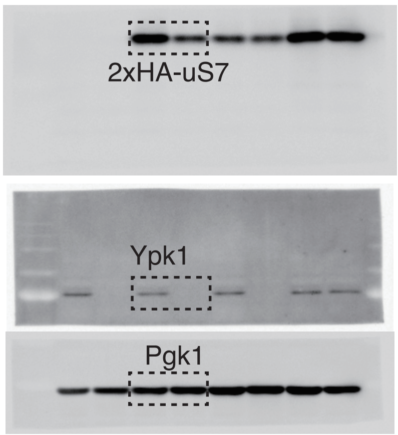

Figure 1C

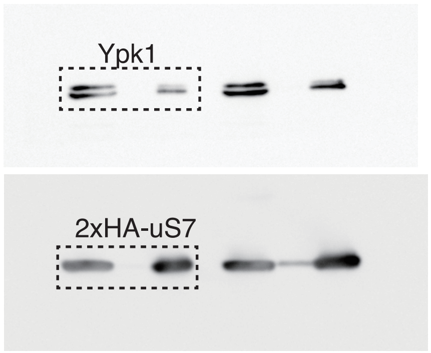

Figure 1D

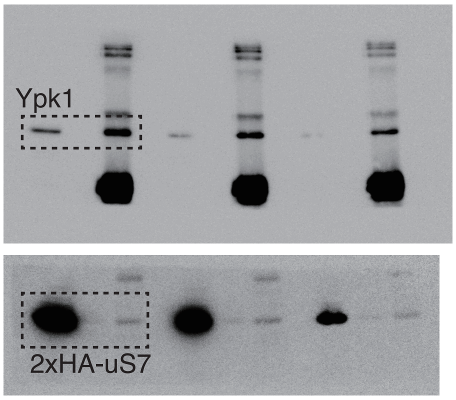

Figure 2A

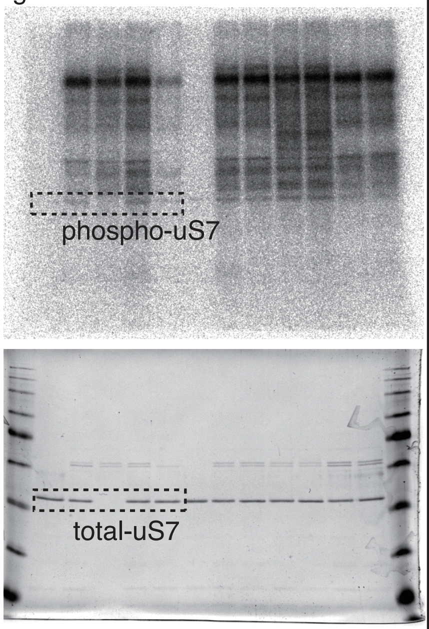

Figure 2D

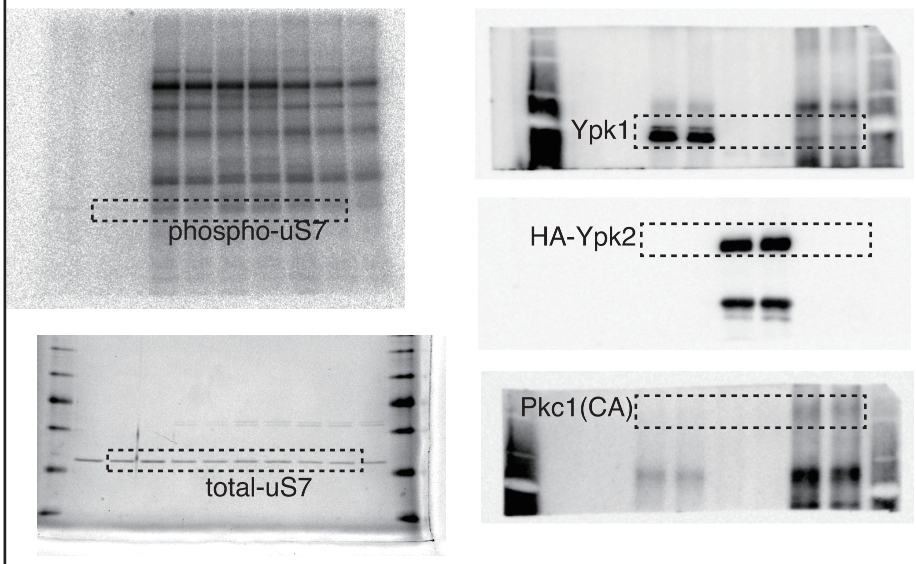

Figure 4A

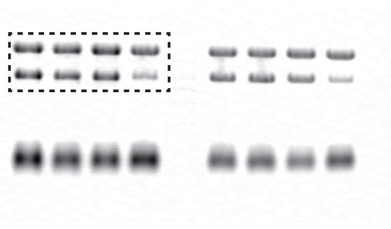

Figure 4C

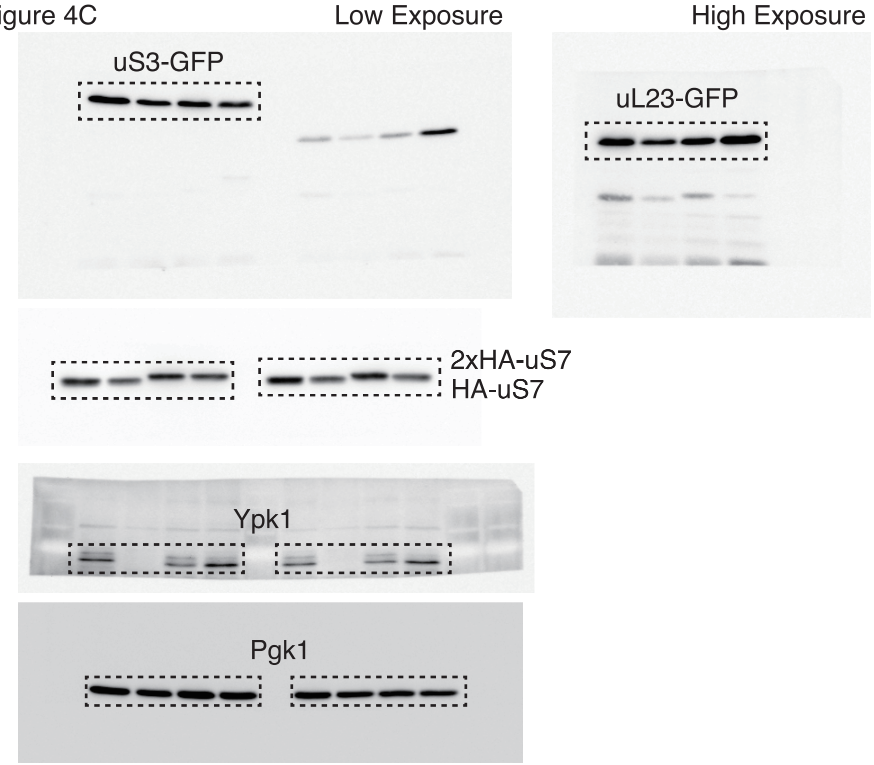

Supplemental Gel Images 2

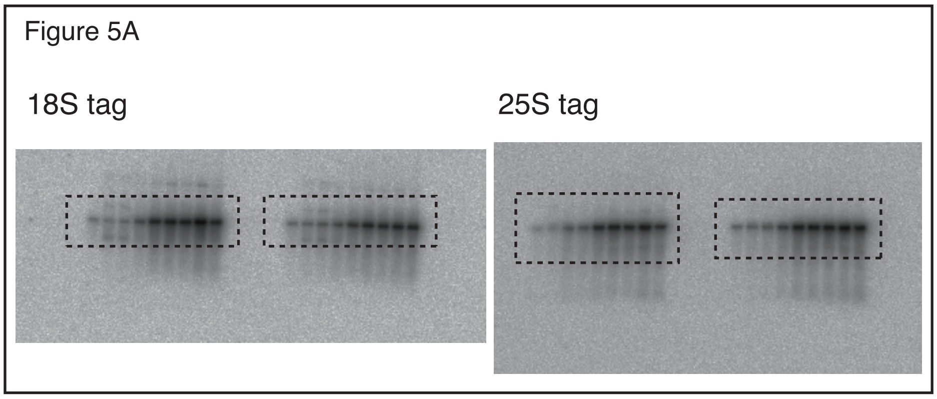

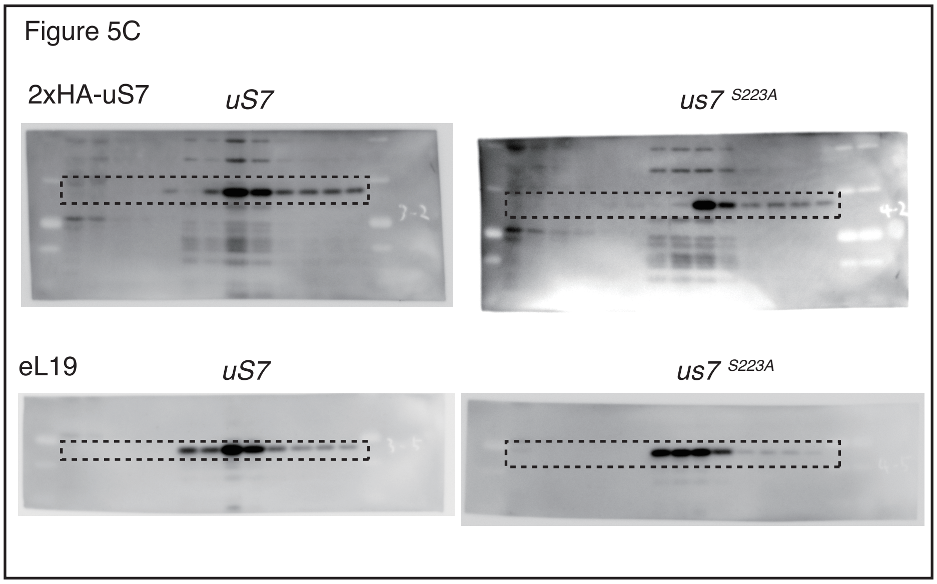

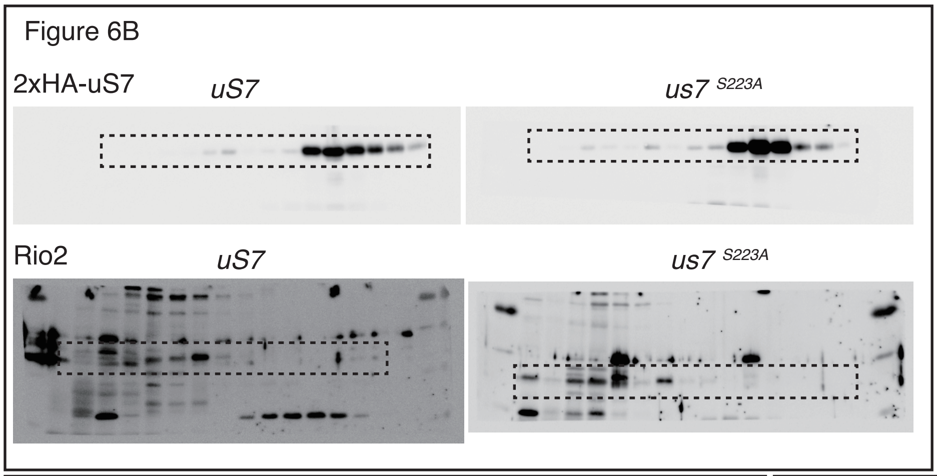

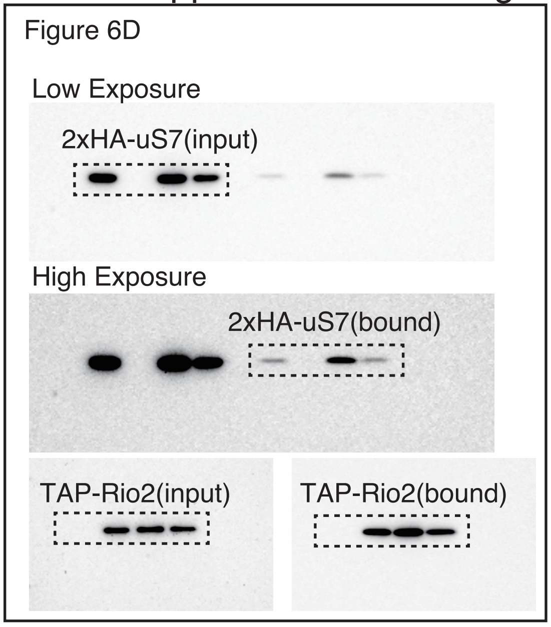

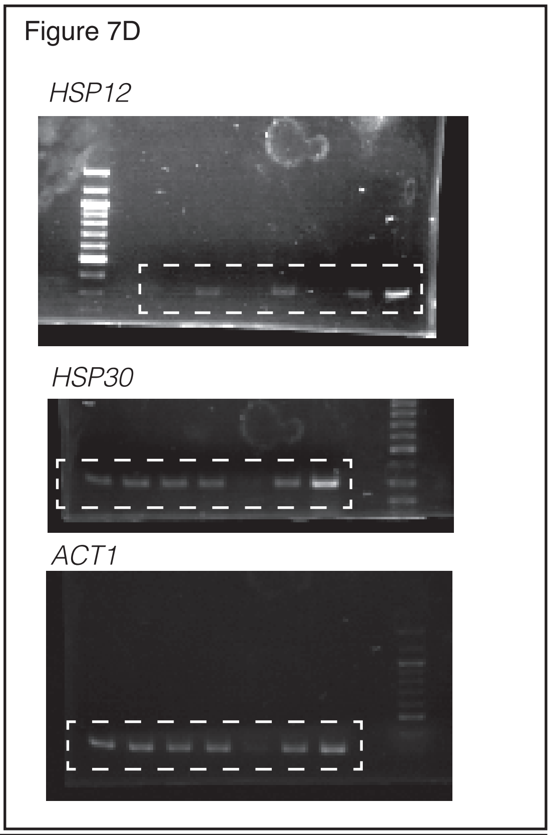

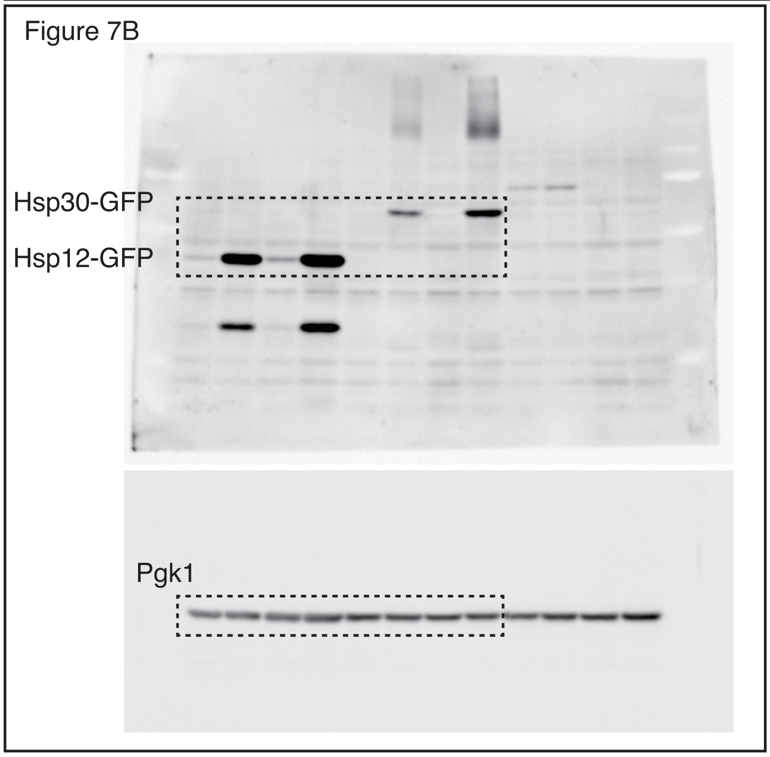

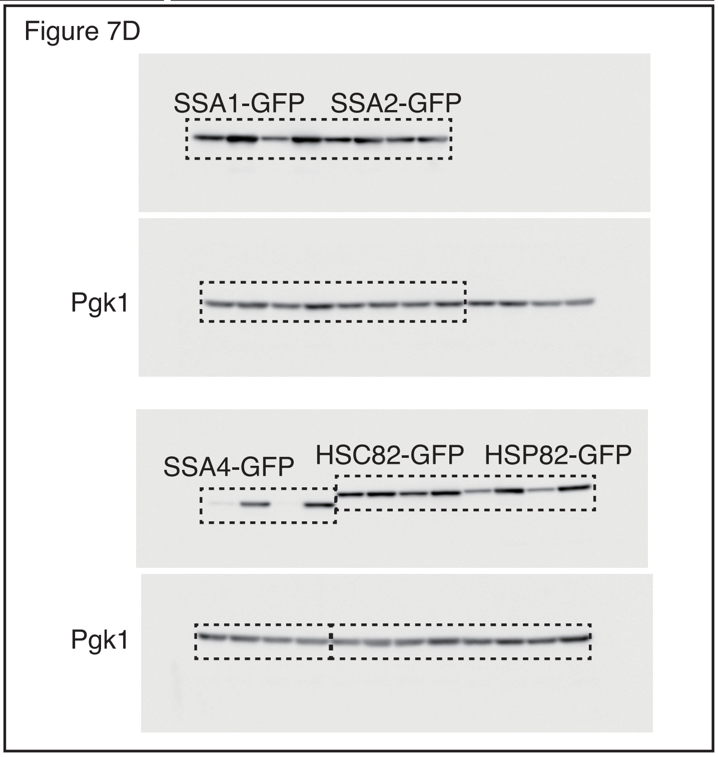

Supplement: Supplementary file 1 — Supplemental Information [file 41598_2018_19652_MOESM1_ESM.pdf]
